# Establishment of the Korea National Health and Nutrition Examination Survey air pollution study dataset for the researchers on the health impact of ambient air pollution

**DOI:** 10.4178/epih.e2021015

**Published:** 2021-02-08

**Authors:** Myung-Jae Hwang, Jisun Sung, Miryoung Yoon, Jong-Hun Kim, Hui-Young Yun, Dae-Ryun Choi, Youn-Seo Koo, Kyungwon Oh, Sungha Yun, Hae-Kwan Cheong

**Affiliations:** 1 Department of Social and Preventive Medicine, Sungkyunkwan University School of Medicine, Suwon, Korea; 2 Department of Environmental and Energy Engineering, Anyang University, Anyang, Korea; 3 Division of Health and Nutrition Survey and Analysis, Bureau of Chronic Disease Prevention and Control, Korea Disease Control and Prevention Agency, Cheongju, Korea

**Keywords:** Korea National Health and Nutrition Examination Survey, Air pollution, Particulate matter, Health impact, Data profile

## Abstract

To provide a nationwide representative dataset for the study on health impact of air pollution, we combined the data from the Korea National Health and Nutrition Examination Survey with the daily air quality and weather data by matching the date of examination and the residential address of the participants. The database of meteorological factors and air quality as sources of exposure data were estimated using the Community Multiscale Air Quality model. The linkage dataset was merged by three ways; administrative district, *si-gun-gu* (city, county, and district), and geocode (in latitude and longitude coordinate units) based on the participants’ residential address, respectively. During the study period, the exposure dataset of 85,018 individuals (38,306 men and 46,712 women) whose examination dates were recorded were obtained. According to the definition of exposure period, the dataset was combined with the data on short-term, mid-term, and long-term exposure to air pollutants and the meteorological indices. Calculation of the daily merged dataset’s average air pollution linked by *si-gun-gu* and geocode units showed similar results. This study generated a daily average of meteorological indices and air pollution exposure dataset for all regions including rural and remote areas in Korea for 11 years. It is expected to provide a platform for the researchers studying the health impact of air pollution and climate change on the representative population and area, which may facilitate the establishment of local health care plans by understanding the residents’ health status at the local as well as national level.

## INTRODUCTION

Air pollution, which annually causes over 4.2 million premature deaths worldwide, has emerged as one of the major public health hazards [1]. Since the 1960s, air pollution in Korea has been intensified due to rapid urbanization and industrialization. Studies on the health impact of ambient air pollution has been reported since the late 1990s, but most of them were conducted mainly in Seoul or metropolitan areas; thus, there is a lack of nationwide research evaluating the health impact of air pollution, especially in the rural areas. Compared with countries like the United States or China, Korea has smaller land area and higher population density, which lead to air pollution affecting the whole country. Therefore, research on the health impact of air pollution in Korea should be carried out nationwide, taking into account the demographic and socioeconomic differences among regions.

The number of air pollution monitoring posts has recently been increased, but there is still lack of monitoring posts in areas other than the metropolitan areas and capital region [2-4]. In particular, the ultrafine particles, which emerged as a major public concern lately, have been monitored only in the capital region, greatly hindering the conduct of a nationwide research on health impact of air pollution.

The Korea Disease Control and Prevention Agency produces health database (DB) by conducting a systematic national survey including the Korea National Health and Nutrition Examination Survey (KNHANES), and the cohort study, the Korean Genome and Epidemiology Study, on a regular basis [6]. These DBs have strengths in their nationwide coverage and providing diverse information on the regional differences over decades. It also have diverse information on health and socioeconomic status of the population, and implemented with strict quality control system, meeting the global standard as a data source for conducting an outstanding health research. When these DBs are combined with air pollution dataset, they can be applied immediately to studies on the health impact of air pollution [5]. To enable the conduct of a national-level research, which links weather/air quality DB with health DB, the spatiotemporal compatibility of those two datasets must be obtained and the spatiotemporal resolution and the range of dataset must be identical in order for them to be merged.

For assessment of health impact of air pollution, researchers are struggling to acquire a data source to determine the actual exposure date (date of survey, date of examination, etc.), atmospheric concentration of air pollutants on the same date, and residential address of the participants on si-gun-gu (city, county, or district), which are all necessary for examining the health impact of exposure to air pollution. Individual researchers used to have a restricted access in merging each participant’s health DB since it is only provided in low resolution for the purpose of privacy protection. Institutions which has a legal authority must create an aggregate DB by converting the existing data into anonymous, low resolution dataset and directly linking the existing health DB and environmental DB. The institution can then provide the dataset to individual researchers without having access to the personal information or spatiotemporal information of the individual participants during the conduct of epidemiological research.

This project has established environmental hazard factor DB including air pollution DB in systematic methods and created a linkage DB with KNHANES. This project aimed to provide a DB platform to researchers, expecting to nurture active research, providing a scientific ground for establishing healthcare plans at the local as well as national government level.

## DATA RESOURCE

The KNHANES is a nationwide survey conducted based on Article 16 of National Health Promotion Act, which was established in 1995. It had been conducted every 3 years between 1998 and 2005, and annually after 2007. KNHANES has been producing statistical data, which showed national-level, metropolitan city-level, and provincial-level representativeness and credibility on the status of health including physical examination and laboratory testing, health-related awareness and behavior, and dietary and nutritional intake (Table 1) [6,7].

Health interview survey and health examination survey were both conducted in mobile examination centers, and nutrition survey was conducted via household visits. Data on socioeconomic status (e.g., education and economic activity), morbidity, medical use, and other items in the nutrition survey were obtained through interview, while health behaviors such as cigarette smoking and alcohol use were examined using self-administered questionnaire. All criteria for health examination were fulfilled through direct measurement, observation, and subject analysis, etc.

KNHANES uses the most updated dataset of population and housing census from the point of sample construction for the sampling frame and complements the basic sampling frame by adding the dataset for declared value of multi-unit houses, which provides the latest information reflecting the population features, thus improving the population coverage rate. This enables the KNHANES to obtain the representative sample from the target population, which consists of citizens aged 1 year and older, currently residing in Korea. The basic sampling frames for KNHANES are the population and housing census, but the KNHANES V dataset (2010-2012) was replaced with the data of the survey on registered population and market price of apartment housing because it was already outdated [7].

### Population coverage

This project linked the exposure data of 85,018 participants whose examination dates have been recorded, among the total study population who participated in the survey between 2007 and 2017. Examination of the distribution of the annual number of survey participants showed that the number was lowest in 2007, data of which were from the KNHANES IV (n= 4,246), since the survey was conducted only from July to December in 2007 and the number of participants was greatest in 2009 (n= 10,078). The regional distribution in the 17 metropolitan cities and provinces showed that Gyeonggi-do (province) had the biggest participants (n= 16,181), while Sejong Metropolitan city, which was only included in the survey in 2016, had the smallest participants (n= 330) (Table 2).

Demographic characteristics of the participants of the KNHANES 2007-2017 were as follows: There were more women, 46,712 (54.9%), compared to the mens, 38,306 (45.1%). The subgroup that participated most was age group above 50 and below 60 (n= 12,107), monthly income between 2 million Korean won and 3 million Korean won (n= 23,981), and educational level of elementary school or below (n= 32,138) (Table 3).

### Ethics statement

This present study was approved by the Institutional Review Board (IRB) of Sungkyunkwan University (IRB No. 2019-03-014).

## MEASUREMENTS

### Exposure data

This project created a meteorologic and air quality DB utilizing a chemical transport model, the Community Multiscale Air Quality (CMAQ) model. The meteorologic dataset was created threedimensionally based on temporal unit and grid unit used as input data for emission quantity model and chemical transport model along with wind field, temperature field, and humidity field. Based on the meteorologic dataset and the characteristics of each emission source, this emission quantity model generates emission quantity depending on the chemical speciation and spatiotemporal allocation, which can be applied to the air quality model.

The chemical transport model uses a meteorologic factor of the meteorologic dataset and numerically interprets the chemical reaction of gas and aerosol, and the advection-diffusion equation is used to spatiotemporally estimate the pollutant concentration. We entered the meteorologic reanalysis data generated via CMAQ to the Weather Research Forecast version 3.6.1 and used Sparse Matrix Operator Kernel Emissions version 2.7 for the emission quantity generation model [8].

The calculated meteorologic data included daily average temperature, average humidity, average precipitation, average wind direction, average solar radiation, and average surface pressure, which were measured in every 3 km. As for the air quality data, particulate matter (PM_10_) and ultrafine particulate matter (PM_2.5_) were measured every 1 km^2^ (1 km× 1 km), while gaseous materials such as nitrogen dioxide (NO_2_), carbon monoxide (CO), sulfur dioxide (SO_2_), and ozone (O_3_) were measured every 9 km^2^ (3 km× 3 km). The data were basically generated as a geocode, and the local exposure data were calculated by reassessing the gridded data as a weighted sum according to the boundaries of the city (*si*), county (*gun*), and district (*gu*).

This data complemented and verified the results of the reanalysis of PM_10_, PM_2.5_, and O_3_ using the satellite observed data of aerosol optical depth and applying the multiple linear regression method. Since there is a lack of measurement data on PM_2.5_ before 2013, we calculated the reanalysis data based on the performance results of the chemical transport model. As a result of reanalyzing the model data and verifying the correlation and validity with the measurement from the actual observation point, PM_10_ and PM_2.5_ showed R^2^ values of 0.81 and 0.64, respectively [8].

### Definition of exposure period

In general, assessment of the effect of air pollution on the morbidity and mortality is conducted through a time-series study [9,10]. Three categories of exposure period—short-term, midterm, and long-term—are observable when evaluating the health impact of exposure to air pollution using time-series data. For the analysis, health impact and exposure period should be matched both in temporal and spatial dimension. Air pollutants absorbed into the body due to short-term exposure has an acute effect on the body’s metabolic mechanism, and continuous exposure (long-term exposure) affects the occurrence and morbidity of the disease, leading to early death.

**Figure 1.**
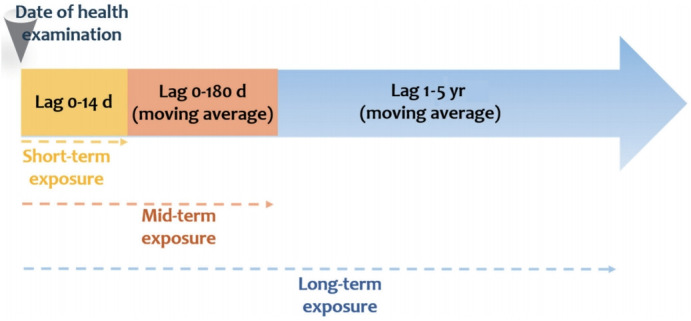
Definition of exposure period in this study.

Therefore, this project organized the daily air pollution DB based on the exposure period so that it can be utilized for various health data provided by the KNHANES depending on the various time span of exposure and health outcome. At the moment, there is no clear-cut definition for air pollution exposure period; hence, we defined it based on the results of studies on the health impact of exposure to environmental hazard factors that have been reported on the literature (Figure 1).

#### Definition of short-term exposure

Numerous epidemiological studies are evaluating the health impact of exposure to highly concentrated air pollution within a short period of time. Previous studies only evaluated the short-term effect of air pollution due to limitations of the health data resource. Recently, studies on the health impact of long-term exposure to air pollution have been actively conducted as access to various sources has become accessible [11-13].

This project linked the short-term data of exposure to air pollution using the examination date of the participants in the KNHANES from 2007 to 2017, which provides both exposure data and health survey data. We linked the daily average meteorological and air quality data from the date of survey, 1 day prior (lag01) to the survey, 2 days prior (lag02) to the survey, …, up to 14 days prior (lag14) to the survey, considering the lag effect of air pollution.

#### Definition of mid-term exposure

The effect of exposure to air pollution is generally divided into short-term exposure and long-term exposure; recently, the number of studies on health impact from mid-term exposure to air pollution has gradually increased as data on various blood biomarkers have become available in examination surveys. For example, taking into account the fact that the life expectancy of normal red blood cells and glycated hemoglobin are approximately 120 days and 60-90 days, respectively, the mid-term exposure must be monitored in order to observe any changes in blood biomarkers due to exposure to air pollution [14].

The mid-term exposure data were obtained by calculating the daily moving average of the meteorological data and air quality data from 30 days, 60 days, 90 days, 120 days, 150 days, and 180 days prior to the date of examination, considering the lag effect.

#### Definition of long-term exposure

In general, long-term exposure to air pollution is assessed based on the annual exposure concentration. This can be utilized not only for long-term follow-up cohort studies to evaluate the health impact of continuous exposure to air pollution, but also for cross-sectional studies such as the KNHANES or Community Health Survey, which have been conducted over years to decades [2,15].

The long-term exposure data were determined by calculating the daily moving average of the meteorological data and air quality data from 365 days (1 year), 730 days (2 years), 1,095 days (3 years), 1,460 days (4 years), and 1,826 days (5 years) prior to the date of examination, considering the lag effect. The moving average data from 5 years prior to the date of examination was calculated because one of that timepoints had a total of 366 days instead of 365 days.

### Merging method

**Figure 2.**
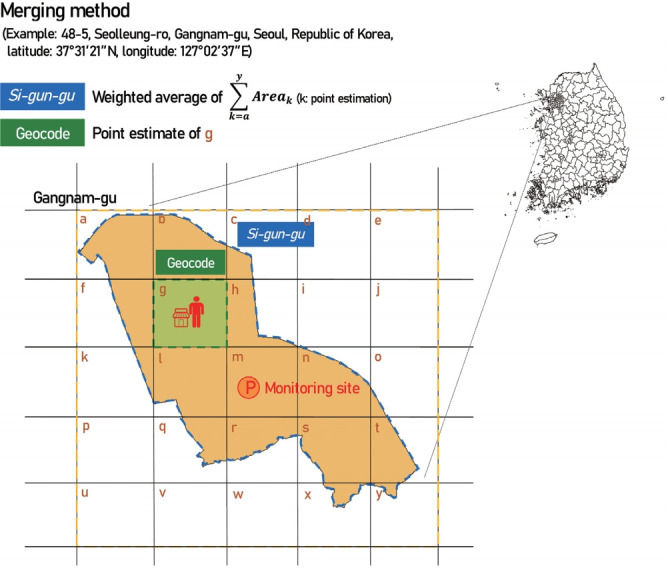
Merging the method for air pollution database and Korea National Health and Nutrition Examination Survey.

In this project, the KNHANES DB was merged with the meteorological DB and air quality DB based on the survey participants’ date of examination. There are two types of linkage data based on the survey participants’ residential address: data merged by *si-gun-gu* (city, county, or district) units and data merged by latitude and longitude (geocode) units (Figure 2) (Supplementary Material 1). These two exposure resolution variables were connected to the examination date variable and together they created a linkage key.

Table 4 shows the distribution of average daily exposure data merged based on the KNHANES participants’ residential address using the meteorological and air quality DB obtained from 2007 to 2017. The average air pollutant exposure level in both data merged by *si-gun-gu* units and data merged by geocode units were comparable. However, the average daily maximum exposure level for certain air pollutants were higher in the data merged by geocode units. The results of mid-term and long-term exposure levels are presented in Supplementary Materials 2-6.

## STRENGTHS AND WEAKNESSES

This project linked the KNHANES and weather/air quality DB to promote the utilization of national health data for research on the health impact of air pollution. Previous studies using national health data to analyze air pollution exposure could only produce results focused mainly on eight metropolitan areas due to the lack of measure point data. To overcome this constraint, this project produced the nationwide weather and air pollution exposure dataset and established the infrastructure to produce a nationally representative research result by enabling the linkage analysis of domestic health data resource such as KNHANES not only for the residents in cities but also for those in rural areas. Moreover, long-term data without seasonal bias could be established as the KNHANES is a survey conducted throughout the year, and thus spatiotemporal representativeness was obtained. Notably, health risks could be evaluated by determining the factors that contribute to the development of diseases caused by exposure to air pollution since information about health behavioral factors such as smoking, drinking, and physical activity on an individual level are provided. We anticipate that the local governments will be more attentive to the health impact on residents and apply the results of this analysis on the local healthcare plans. Nonetheless, there are still limitations such as the disparity between the actual observed value and the value estimated by the weather/air quality DB since they are data drawn from models, and fully overcoming the uncertainty of the non-observed points seems implausible.

KNHANES provides diverse indices related to healthcare by conducting an annual survey and national health indices by conducting a precise examination survey. We linked these domestic representative survey data and air pollution exposure data and prepared a data profile so that epidemiological researchers can utilize the information more efficiently. If these data can be applied in various sectors through this data profile, they will serve as a theoretical ground for establishing and practicing policies related to hazardous air pollution in the local government level by allowing them to conduct comprehensive research on the health impact, reflecting the local demographic and socioeconomic features.

## DATA ACCESSIBILITY

The weather/air pollution DB linked with the KNHANES conducted from 2007 to 2017 will be provided in accordance with the provision procedure for data (http://knhanes.cdc.go.kr). Linkage data will be provided through the Data Processing Center for Academic Research after reviewing the research proposal.

## Figures and Tables

**Table 1.** Survey components of the Korea National Health and Nutrition Examination Survey from 2007 to 2017

Survey	Components
Health interview	Household characteristics, socioeconomic status (e.g., education, household income, and occupational status), economic status, medical use, vaccination, medical conditions, activity limitation, quality of life, injury, smoking, alcohol use, physical activity, mental health, oral health, safety, weight control, and reproductive health for women
Health examination	Body measurement, blood pressure and pulse rate, blood test, urine test, muscle strength test, lung function, dental examination, otolaryngeal and ophthalmologic examination
Nutrition survey	Dietary behavior, dietary supplement use, households with food security, food frequency, and food intake

**Table 2.** Distribution of survey participants of the 2007-2017 Korea National Health and Nutrition Examination Survey in 17 regions^1^

Regions	Year											Total
	2007	2008	2009	2010	2011	2012	2013	2014	2015	2016	2017	
Metropolitan city												
Seoul	708	1,543	1,680	1,705	1,647	1,618	1,455	1,335	1,313	1,526	1,484	16,014
Busan	241	596	633	482	497	425	388	423	348	500	479	5,012
Daegu	245	427	467	408	384	384	440	406	405	370	368	4,304
Incheon	195	564	648	483	488	442	452	410	451	419	477	5,029
Gwangju	160	395	348	234	258	237	229	218	235	244	253	2,811
Daejeon	142	301	354	277	223	249	270	299	252	265	255	2,887
Ulsan	88	249	254	198	201	196	164	145	136	144	172	1,947
Sejong^2^	NA	NA	NA	NA	NA	NA	NA	NA	NA	164	166	330
Province												
Gyeonggi-do	854	1,930	2,104	2,003	1,845	1,713	1,906	1,781	1,660	201	184	16,181
Gangwon-do	148	290	341	257	221	221	249	251	290	1,830	1,804	5,902
Chungcheongbuk-do	122	333	348	236	272	194	214	231	198	220	252	2,620
Chungcheongnam-do	226	419	514	368	325	310	316	282	316	259	207	3,542
Jeollabuk-do	135	406	351	328	316	309	245	222	257	315	239	3,123
Jeollanam-do	220	481	472	321	313	304	206	187	220	252	294	3,270
Gyeongsangbuk-do	342	601	672	518	429	459	438	393	377	263	253	4,745
Gyeongsangnam-do	308	549	624	475	456	425	421	427	374	387	386	4,832
Jeju-do	112	223	268	180	172	154	178	157	145	444	436	2,469
Total	4,246	9,307	10,078	8,473	8,047	7,640	7,571	7,167	6,977	7,803	7,709	85,018

NA, not applicable.

1 Evaluation of the socio-demographic characteristics showed that the women participants outnumbered the men participants by 9.8% point (women: n=46,712, 54.9% and men: n=38,306, 45.1%). The group aged 50-60 years had the highest number of participants (n=12,107), followed by those aged 40-50 years and those aged 30-40 years. Over 1,151 participants did not respond to the question related to income level. Meanwhile, 23,981 participants responded to the question related to the income level: “above 2 million Korean won and below 3 million Korean won.” A total of 3,495 participants did not respond to the question related to education level, while 32,138 responded that they only finished “elementary school.”

2 Survey was included in the survey in 2016.

**Table 3.** Demographic characteristics of participants in the 2007-2017 Korea National Health and Nutrition Examination Survey

Characteristics	Men	Women	Total
Total	38,306 (45.1)	46,712 (54.9)	85,018
Age (yr)			
1-9	5,340 (6.3)	4,920 (5.8)	10,260
10-19	5,286 (6.2)	4,797 (5.6)	10,083
20-29	3,106 (3.7)	4,095 (4.8)	7,201
30-39	4,891 (5.8)	6,764 (8.0)	11,655
40-49	5,285 (6.2)	6,811 (8.0)	12,096
50-59	5,152 (6.1)	6,955 (8.2)	12,107
60-69	4,866 (5.6)	6,097 (7.2)	10,963
≥70	4,380 (5.1)	6,273 (7.4)	10,653
Income level (unit: 10 thousand Korean won)			
≤100	6,036 (7.0)	8,909 (10.6)	14,945
100-200	9,668 (11.4)	11,833 (13.9)	21,501
200-300	11,126 (13.1)	12,855 (15.1)	23,981
>300	10,993 (12.9)	12,447 (14.6)	23,440
Missing	483 (0.6)	668 (0.8)	1,151
Education level			
Elementary school	13,279 (15.6)	18,859 (22.2)	32,138
Middle school	4,436 (5.1)	4,922 (5.8)	9,358
High school	9,654 (11.4)	11,442 (13.5)	21,096
Over university	9,241 (10.9)	9,690 (11.4)	18,931
Missing	1,696 (2.0)	1,799 (2.1)	3,495

Values are presented as number (%) or number.

**Table 4.** Daily exposure level of ambient air pollutants during the study period (2007-2017)

Air pollutants	Mean	SD	Min	Percentile			Max	IQR
				25th	50th	75th		
Si-gun-gu								
PM_10_ (μg/m^3^)	49.6	21.6	10.9	34.6	45.9	59.8	260.8	25.2
PM_2.5_ (μg/m^3^)	25.2	11.9	2.2	16.6	23.2	31.4	95.3	14.8
NO_2_ (ppb)	24.4	13.8	0.3	13.6	21.8	32.6	98.3	19.0
CO (ppb)	489.4	219.1	90.8	341.6	439.7	571.8	1,841.4	230.2
SO_2_ (ppb)	4.9	2.7	0.3	3.1	4.3	6.1	30.1	2.9
O_3_ (ppb)	24.7	10.7	0.8	16.5	23.6	32.3	72.7	15.8
Geocode								
PM_10_ (μg/m^3^)	49.8	21.9	10.4	34.4	46.0	60.0	259.5	25.6
PM_2.5_ (μg/m^3^)	25.3	12.1	0.9	16.6	23.1	31.5	90.7	14.9
NO_2_ (ppb)	24.5	14.0	0.3	13.7	21.8	32.8	101.9	19.1
CO (ppb)	490.8	223.4	88.4	342.6	442.2	575.4	1,808.8	232.8
SO_2_ (ppb)	5.0	3.0	0.1	3.1	4.3	6.1	44.5	2.9
O_3_ (ppb)	24.7	10.8	0.8	16.4	23.7	32.5	76.2	16.1

SD, standard deviation; Min, minimum; Max, maximum; IQR, interquartile range; PM_10_, particulate matter; PM_2.5_, ultrafine particulate matter; NO_2_, nitrogen dioxide; CO, carbon monoxide; SO_2_, sulfur dioxide; O_3_, ozone; ppb, parts per billion.

## SUPPLEMENTARY MATERIALS

Supplementary Material 1. Data structure of the KNHANES air pollution dataset

Supplementary Material 2. Exposure level of moving average of 0 to 30 days of ambient air pollutants during the study period (2007-2017)

Supplementary Material 3. Exposure level of moving average of 0 to 90 days of ambient air pollutants during the study period (2007-2017)

Supplementary Material 4. Exposure level of moving average of 0 to 180 days of ambient air pollutants during the study period (2007-2017)

Supplementary Material 5. Exposure level of moving average of 0 to 365 days of ambient air pollutants during the study period (2007-2017)

Supplementary Material 6. Exposure level of moving average of 0 to 1,095 days of ambient air pollutants during the study period (2007-2017)

Korean version is available at http://www.e-epih.org/.
