# Supplementary material for: Establishment of the Korea National Health and Nutrition Examination Survey air pollution study dataset for the researchers on the health impact of ambient air pollution
Source: Epidemiol Health. 2021 Feb 8;43:e2021015. doi: 10.4178/epih.e2021015 (PMC8060520; doi:10.4178/epih.e2021015)
Supplement: Supplementary Material 1. — Data structure of the KNHANES air pollution dataset [file epih-43-e2021015-suppl1.pdf]

Volume: 43, Article ID: e2021015

https://doi.org/10.4178/epih.e2021015

Supplemental Material 1. Data structure of the KNHANES air pollution dataset

Geocode	Si-gun-gu	Description	Format	Unit	Remarks
NEW_ID		ID	문자		
NEW_ID_FAM		가구 ID			
NEW_PSU		PSU			
TEMP	si_TEMP	당일의 평균기온	숫자	℃	노출자료연계는 검진조사일자를 기준으로 매칭
t_lag01	si_t_lag01	1일전 평균기온	숫자	℃	
t_lag02	si_t_lag02	2일전 평균기온	숫자	℃	
t_lag03	si_t_lag03	3일전 평균기온	숫자	℃	
t_lag04	si_t_lag04	4일전 평균기온	숫자	℃	
t_lag05	si_t_lag05	5일전 평균기온	숫자	℃	
t_lag06	si_t_lag06	6일전 평균기온	숫자	℃	
t_lag07	si_t_lag07	7일전 평균기온	숫자	℃	
t_lag08	si_t_lag08	8일전 평균기온	숫자	℃	
t_lag09	si_t_lag09	9일전 평균기온	숫자	℃	
t_lag10	si_t_lag10	10일전 평균기온	숫자	℃	
t_lag11	si_t_lag11	11일전 평균기온	숫자	℃	
t_lag12	si_t_lag12	12일전 평균기온	숫자	℃	
t_lag13	si_t_lag13	13일전 평균기온	숫자	℃	
t_lag14	si_t_lag14	14일전 평균기온	숫자	℃	
t_ma30	si_t_ma30	0-30일전까지의 이동평균기온	숫자	℃	

t_ma60	si_t_ma60	0-60일전까지의 이동평균기온	숫자	℃	
t_ma90	si_t_ma90	0-90일전까지의 이동평균기온	숫자	℃	
t_ma120	si_t_ma120	0-120일전까지의 이동평균기온	숫자	℃	
t_ma150	si_t_ma150	0-150일전까지의 이동평균기온	숫자	℃	
t_ma180	si_t_ma180	0-180일전까지의 이동평균기온	숫자	℃	
t_lag1y	si_t_lag1y	0-365일전까지의 이동평균기온	숫자	℃	
t_lag2y	si_t_lag2y	0-730일전까지의 이동평균기온	숫자	℃	
t_lag3y	si_t_lag3y	0-1,095일전까지의 이동평균기온	숫자	℃	
t_lag4y	si_t_lag4y	0-1,460일전까지의 이동평균기온	숫자	℃	
t_lag5y	si_t_lag5y	0-1,826일전까지의 이동평균기온	숫자	℃	366일은 감안하여 5년을 1,826일로 계산함
WS	si_WS	당일의 평균풍속	숫자	m/s	
ws_lag01	si_ws_lag01	1일전 평균풍속	숫자	m/s	
ws_lag02	si_ws_lag02	2일전 평균풍속	숫자	m/s	
ws_lag03	si_ws_lag03	3일전 평균풍속	숫자	m/s	
ws_lag04	si_ws_lag04	4일전 평균풍속	숫자	m/s	
ws_lag05	si_ws_lag05	5일전 평균풍속	숫자	m/s	
ws_lag06	si_ws_lag06	6일전 평균풍속	숫자	m/s	
ws_lag07	si_ws_lag07	7일전 평균풍속	숫자	m/s	
ws_lag08	si_ws_lag08	8일전 평균풍속	숫자	m/s	
ws_lag09	si_ws_lag09	9일전 평균풍속	숫자	m/s	

Volume: 43, Article ID: e2021015

https://doi.org/10.4178/epih.e2021015

ws_lag10	si_ws_lag10	10일전 평균풍속	숫자	m/s	
ws_lag11	si_ws_lag11	11일전 평균풍속	숫자	m/s	
ws_lag12	si_ws_lag12	12일전 평균풍속	숫자	m/s	
ws_lag13	si_ws_lag13	13일전 평균풍속	숫자	m/s	
ws_lag14	si_ws_lag14	14일전 평균풍속	숫자	m/s	
ws_ma30	si_ws_ma30	0-30일전까지의 평균풍속	숫자	m/s	
ws_ma60	si_ws_ma60	0-60일전까지의 평균풍속	숫자	m/s	
ws_ma90	si_ws_ma90	0-90일전까지의 평균풍속	숫자	m/s	
ws_ma120	si_ws_ma120	0-120일전까지의 평균풍속	숫자	m/s	
ws_ma150	si_ws_ma150	0-150일전까지의 평균풍속	숫자	m/s	
ws_ma180	si_ws_ma180	0-180일전까지의 평균풍속	숫자	m/s	
ws_lag1y	si_ws_lag1y	0-365일전까지의 이동평균풍속	숫자	m/s	
ws_lag2y	si_ws_lag2y	0-730일전까지의 이동평균풍속	숫자	m/s	
ws_lag3y	si_ws_lag3y	0-1,095일전까지의 이동평균풍속	숫자	m/s	
ws_lag4y	si_ws_lag4y	0-1,460일전까지의 이동평균풍속	숫자	m/s	
ws_lag5y	si_ws_lag5y	0-1,826일전까지의 이동평균풍속	숫자	m/s	366일을 감안하여 5년은 1,826일로 계산함
RH	si_RH	당일의 평균습도	숫자	%	
rh_lag01	si_rh_lag01	1일전 평균습도	숫자	%	
rh_lag02	si_rh_lag02	2일전 평균습도	숫자	%	
rh_lag03	si_rh_lag03	3일전 평균습도	숫자	%	

Volume: 43, Article ID: e2021015

https://doi.org/10.4178/epih.e2021015

rh_lag04	si_rh_lag04	4일전 평균습도	숫자	%
rh_lag05	si_rh_lag05	5일전 평균습도	숫자	%
rh_lag06	si_rh_lag06	6일전 평균습도	숫자	%
rh_lag07	si_rh_lag07	7일전 평균습도	숫자	%
rh_lag08	si_rh_lag08	8일전 평균습도	숫자	%
rh_lag09	si_rh_lag09	9일전 평균습도	숫자	%
rh_lag10	si_rh_lag10	10일전 평균습도	숫자	%
rh_lag11	si_rh_lag11	11일전 평균습도	숫자	%
rh_lag12	si_rh_lag12	12일전 평균습도	숫자	%
rh_lag13	si_rh_lag13	13일전 평균습도	숫자	%
rh_lag14	si_rh_lag14	14일전 평균습도	숫자	%
rh_ma30	si_rh_ma30	0-30일전까의 이동평균습도	숫자	%
rh_ma60	si_rh_ma60	0-60일전까지의 이동평균습도	숫자	%
rh_ma90	si_rh_ma90	0-90일전까지의 이동평균습도	숫자	%
rh_ma120	si_rh_ma120	0-120일전까지의 이동평균습도	숫자	%
rh_ma150	si_rh_ma150	0-150일전까지의 이동평균습도	숫자	%
rh_ma180	si_rh_ma180	0-180일전까지의 이동평균습도	숫자	%
rh_lag1y	si_rh_lag1y	0-365일전까지의 이동평균습도	숫자	%
rh_lag2y	si_rh_lag2y	0-730일전까지의 이동평균습도	숫자	%
rh_lag3y	si_rh_lag3y	0-1,095일전까지의 이동평균습도	숫자	%
rh_lag4y	si_rh_lag4y	0-1,460일전까지의	숫자	%

Volume: 43, Article ID: e2021015

https://doi.org/10.4178/epih.e2021015

		이동평균습도			
rh_lag5y	si_rh_lag5y	0-1,826일전까지의 이동평균습도	숫자	%	
RN	si_RN	당일의 평균강수량	숫자	cm/hr	*24시간 평균농도이므로 24를 곱하여 누적강수량으로 사용할 수 없음.
rn_lag01	si_rn_lag01	1일전 평균강수량	숫자	cm/hr	*24시간 평균농도이므로 24를 곱하여 누적강수량으로 사용할 수 없음.
rn_lag02	si_rn_lag02	2일전 평균강수량	숫자	cm/hr	*24시간 평균농도이므로 24를 곱하여 누적강수량으로 사용할 수 없음.
rn_lag03	si_rn_lag03	3일전 평균강수량	숫자	cm/hr	*24시간 평균농도이므로 24를 곱하여 누적강수량으로 사용할 수 없음.
rn_lag04	si_rn_lag04	4일전 평균강수량	숫자	cm/hr	*24시간 평균농도이므로 24를 곱하여 누적강수량으로 사용할 수 없음.
rn_lag05	si_rn_lag05	5일전 평균강수량	숫자	cm/hr	*24시간 평균농도이므로 24를 곱하여 누적강수량으로 사용할 수 없음.
rn_lag06	si_rn_lag06	6일전 평균강수량	숫자	cm/hr	*24시간 평균농도이므로 24를 곱하여 누적강수량으로 사용할 수 없음.
rn_lag07	si_rn_lag07	7일전 평균강수량	숫자	cm/hr	*24시간 평균농도이므로 24를 곱하여 누적강수량으로 사용할 수 없음.
rn_lag08	si_rn_lag08	8일전 평균강수량	숫자	cm/hr	*24시간 평균농도이므로 24를 곱하여 누적강수량으로 사용할 수

Volume: 43, Article ID: e2021015

https://doi.org/10.4178/epih.e2021015

					없음.
rn_lag09	si_rn_lag09	9일전 평균강수량	숫자	cm/hr	*24시간 평균농도이므로 24를 곱하여 누적강수량으로 사용할 수 없음.
rn_lag10	si_rn_lag10	10일전 평균강수량	숫자	cm/hr	*24시간 평균농도이므로 24를 곱하여 누적강수량으로 사용할 수 없음.
rn_lag11	si_rn_lag11	11일전 평균강수량	숫자	cm/hr	*24시간 평균농도이므로 24를 곱하여 누적강수량으로 사용할 수 없음.
rn_lag12	si_rn_lag12	12일전 평균강수량	숫자	cm/hr	*24시간 평균농도이므로 24를 곱하여 누적강수량으로 사용할 수 없음.
rn_lag13	si_rn_lag13	13일전 평균강수량	숫자	cm/hr	*24시간 평균농도이므로 24를 곱하여 누적강수량으로 사용할 수 없음.
rn_lag14	si_rn_lag14	14일전 평균강수량	숫자	cm/hr	*24시간 평균농도이므로 24를 곱하여 누적강수량으로 사용할 수 없음.
rn_ma30	si_rn_ma30	0-30일전까지의 이동평균강수량	숫자	cm/hr	*24시간 평균농도이므로 24를 곱하여 누적강수량으로 사용할 수 없음.
rn_ma60	si_rn_ma60	0-60일전까지의 이동평균강수량	숫자	cm/hr	*24시간 평균농도이므로 24를 곱하여 누적강수량으로 사용할 수 없음.
rn_ma90	si_rn_ma90	0-90일전까지의 이동평균강수량	숫자	cm/hr	*24시간 평균농도이므로 24를 곱하여 누적강수량으로 사용할 수 없음.

Volume: 43, Article ID: e2021015

https://doi.org/10.4178/epih.e2021015

rn_ma120	si_rn_ma120	0-120일전까지의 이동평균강수량	숫자	cm/hr	*24시간 평균농도이므로 24를 곱하여 누적강수량으로 사용할 수 없음.
rn_ma150	si_rn_ma150	0-150일전까지의 이동평균강수량	숫자	cm/hr	*24시간 평균농도이므로 24를 곱하여 누적강수량으로 사용할 수 없음.
rn_ma180	si_rn_ma180	0-180일전까지의 이동평균강수량	숫자	cm/hr	*24시간 평균농도이므로 24를 곱하여 누적강수량으로 사용할 수 없음.
rn_lag1y	si_rn_lag1y	0-365일전까지의 이동평균강수량	숫자	cm/hr	*24시간 평균농도이므로 24를 곱하여 누적강수량으로 사용할 수 없음.
rn_lag2y	si_rn_lag2y	0-730일전까지의 이동평균강수량	숫자	cm/hr	*24시간 평균농도이므로 24를 곱하여 누적강수량으로 사용할 수 없음.
rn_lag3y	si_rn_lag3y	0-1,095일전까지의 이동평균강수량	숫자	cm/hr	*24시간 평균농도이므로 24를 곱하여 누적강수량으로 사용할 수 없음.
rn_lag4y	si_rn_lag4y	0-1,460일전까지의 이동평균강수량	숫자	cm/hr	*24시간 평균농도이므로 24를 곱하여 누적강수량으로 사용할 수 없음.
rn_lag5y	si_rn_lag5y	0-1,826일전까지의 이동평균강수량	숫자	cm/hr	*24시간 평균농도이므로 24를 곱하여 누적강수량으로 사용할 수 없음.
WD	si_WD	당일의 평균풍향	숫자	degree	
wd_lag01	si_wd_lag01	1일전 평균풍향	숫자	degree	
wd_lag02	si_wd_lag02	2일전 평균풍향	숫자	degree	
wd_lag03	si_wd_lag03	3일전 평균풍향	숫자	degree	

Volume: 43, Article ID: e2021015

https://doi.org/10.4178/epih.e2021015

wd_lag04	si_wd_lag04	4일전 평균풍향	숫자	degree
wd_lag05	si_wd_lag05	5일전 평균풍향	숫자	degree
wd_lag06	si_wd_lag06	6일전 평균풍향	숫자	degree
wd_lag07	si_wd_lag07	7일전 평균풍향	숫자	degree
wd_lag08	si_wd_lag08	8일전 평균풍향	숫자	degree
wd_lag09	si_wd_lag09	9일전 평균풍향	숫자	degree
wd_lag10	si_wd_lag10	10일전 평균풍향	숫자	degree
wd_lag11	si_wd_lag11	11일전 평균풍향	숫자	degree
wd_lag12	si_wd_lag12	12일전 평균풍향	숫자	degree
wd_lag13	si_wd_lag13	13일전 평균풍향	숫자	degree
wd_lag14	si_wd_lag14	14일전 평균풍향	숫자	degree
wd_ma30	si_wd_ma30	0-30일전까지의 이동평균풍향	숫자	degree
wd_ma60	si_wd_ma60	0-60일전까지의 이동평균풍향	숫자	degree
wd_ma90	si_wd_ma90	0-90일전까지의 이동평균풍향	숫자	degree
wd_ma120	si_wd_ma120	0-120일전까지의 이동평균풍향	숫자	degree
wd_ma150	si_wd_ma150	0-150일전까지의 이동평균풍향	숫자	degree
wd_ma180	si_wd_ma180	0-180일전까지의 이동평균풍향	숫자	degree
wd_lag1y	si_wd_lag1y	0-365일전까지의 이동평균풍향	숫자	degree
wd_lag2y	si_wd_lag2y	0-730일전까지의 이동평균풍향	숫자	degree
wd_lag3y	si_wd_lag3y	0-1,095일전까지의 이동평균풍향	숫자	degree
wd_lag4y	si_wd_lag4y	0-1,460일전까지의	숫자	degree

		이동평균풍향		
wd_lag5y	si_wd_lag5y	0-1,826일전까지의 이동평균풍향	숫자	degree
GSW	si_GSW	당일의 평균일사량	숫자	W/m^2
s_lag01	si_s_lag01	1일전 평균일사량	숫자	W/m^2
s_lag02	si_s_lag02	2일전 평균일사량	숫자	W/m^2
s_lag03	si_s_lag03	3일전 평균일사량	숫자	W/m^2
s_lag04	si_s_lag04	4일전 평균일사량	숫자	W/m^2
s_lag05	si_s_lag05	5일전 평균일사량	숫자	W/m^2
s_lag06	si_s_lag06	6일전 평균일사량	숫자	W/m^2
s_lag07	si_s_lag07	7일전 평균일사량	숫자	W/m^2
s_lag08	si_s_lag08	8일전 평균일사량	숫자	W/m^2
s_lag09	si_s_lag09	9일전 평균일사량	숫자	W/m^2
s_lag10	si_s_lag10	10일전 평균일사량	숫자	W/m^2
s_lag11	si_s_lag11	11일전 평균일사량	숫자	W/m^2
s_lag12	si_s_lag12	12일전 평균일사량	숫자	W/m^2
s_lag13	si_s_lag13	13일전 평균일사량	숫자	W/m^2
s_lag14	si_s_lag14	14일전 평균일사량	숫자	W/m^2
s_ma30	si_s_ma30	0-30일전까지의 이동평균일사량	숫자	W/m^2
s_ma60	si_s_ma60	0-60일전까지의 이동평균일사량	숫자	W/m^2
s_ma90	si_s_ma90	0-90일전까지의	숫자	W/m^2

Volume: 43, Article ID: e2021015

https://doi.org/10.4178/epih.e2021015

		이동평균일사량		
s_ma120	si_s_ma120	0-120일전까지의 이동평균일사량	숫자	W/m2
s_ma150	si_s_ma150	0-150일전까지의 이동평균일사량	숫자	W/m2
s_ma180	si_s_ma180	0-180일전까지의 이동평균일사량	숫자	W/m2
s_lag1y	si_s_lag1y	0-365일전까지의 이동평균일사량	숫자	W/m2
s_lag2y	si_s_lag2y	0-730일전까지의 이동평균일사량	숫자	W/m2
s_lag3y	si_s_lag3y	0-1,095일전까지의 이동평균일사량	숫자	W/m2
s_lag4y	si_s_lag4y	0-1,460일전까지의 이동평균일사량	숫자	W/m2
s_lag5y	si_s_lag5y	0-1,826일전까지의 이동평균일사량	숫자	W/m2
PRESFC	si_PRESFC	당일의 평균지표압력	숫자	hPa
p_lag01	si_p_lag01	1일전 평균지표압력	숫자	hPa
p_lag02	si_p_lag02	2일전 평균지표압력	숫자	hPa
p_lag03	si_p_lag03	3일전 평균지표압력	숫자	hPa
p_lag04	si_p_lag04	4일전 평균지표압력	숫자	hPa
p_lag05	si_p_lag05	5일전 평균지표압력	숫자	hPa
p_lag06	si_p_lag06	6일전 평균지표압력	숫자	hPa
p_lag07	si_p_lag07	7일전 평균지표압력	숫자	hPa
p_lag08	si_p_lag08	8일전 평균지표압력	숫자	hPa

Volume: 43, Article ID: e2021015

https://doi.org/10.4178/epih.e2021015

p_lag09	si_p_lag09	9일전 평균지표압력	숫자	hPa
p_lag10	si_p_lag10	10일전 평균지표압력	숫자	hPa
p_lag11	si_p_lag11	11일전 평균지표압력	숫자	hPa
p_lag12	si_p_lag12	12일전 평균지표압력	숫자	hPa
p_lag13	si_p_lag13	13일전 평균지표압력	숫자	hPa
p_lag14	si_p_lag14	14일전 평균지표압력	숫자	hPa
p_ma30	si_p_ma30	0-30일전까지의 이동평균지표압력	숫자	hPa
p_ma60	si_p_ma60	0-60일전까지의 이동평균지표압력	숫자	hPa
p_ma90	si_p_ma90	0-90일전까지의 이동평균지표압력	숫자	hPa
p_ma120	si_p_ma120	0-120일전까지의 이동평균지표압력	숫자	hPa
p_ma150	si_p_ma150	0-150일전까지의 이동평균지표압력	숫자	hPa
p_ma180	si_p_ma180	0-180일전까지의 이동평균지표압력	숫자	hPa
p_lag1y	si_p_lag1y	0-365일전까지의 이동평균지표압력	숫자	hPa
p_lag2y	si_p_lag2y	0-730일전까지의 이동평균지표압력	숫자	hPa
p_lag3y	si_p_lag3y	0-1,095일전까지의 이동평균지표압력	숫자	hPa
p_lag4y	si_p_lag4y	0-1,460일전까지의 이동평균지표압력	숫자	hPa

Volume: 43, Article ID: e2021015

https://doi.org/10.4178/epih.e2021015

p_lag5y	si_p_lag5y	0-1,826일전까지의 이동평균지표압력	숫자	hPa
PM10	si_PM10	당일의 평균PM_{10}농도	숫자	㎍/㎥
pm10_lag01	si_pm10_lag01	1일전 평균PM_{10}농도	숫자	㎍/㎥
pm10_lag02	si_pm10_lag02	2일전 평균PM_{10}농도	숫자	㎍/㎥
pm10_lag03	si_pm10_lag03	3일전 평균PM_{10}농도	숫자	㎍/㎥
pm10_lag04	si_pm10_lag04	4일전 평균PM_{10}농도	숫자	㎍/㎥
pm10_lag05	si_pm10_lag05	5일전 평균PM_{10}농도	숫자	㎍/㎥
pm10_lag06	si_pm10_lag06	6일전 평균PM_{10}농도	숫자	㎍/㎥
pm10_lag07	si_pm10_lag07	7일전 평균PM_{10}농도	숫자	㎍/㎥
pm10_lag08	si_pm10_lag08	8일전 평균PM_{10}농도	숫자	㎍/㎥
pm10_lag09	si_pm10_lag09	9일전 평균PM_{10}농도	숫자	㎍/㎥
pm10_lag10	si_pm10_lag10	10일전 평균PM_{10}농도	숫자	㎍/㎥
pm10_lag11	si_pm10_lag11	11일전 평균PM_{10}농도	숫자	㎍/㎥
pm10_lag12	si_pm10_lag12	12일전 평균PM_{10}농도	숫자	㎍/㎥
pm10_lag13	si_pm10_lag13	13일전 평균PM_{10}농도	숫자	㎍/㎥
pm10_lag14	si_pm10_lag14	14일전 평균PM_{10}농도	숫자	㎍/㎥
pm10_ma30	si_pm10_ma30	0-30일전까지의 이동평균PM_{10}농도	숫자	㎍/㎥
pm10_ma60	si_pm10_ma60	0-60일전까지의 이동평균PM_{10}농도	숫자	㎍/㎥
pm10_ma90	si_pm10_ma90	0-90일전까지의 이동평균PM_{10}농도	숫자	㎍/㎥

Volume: 43, Article ID: e2021015

https://doi.org/10.4178/epih.e2021015

pm10_ma120	si_pm10_ma120	0-120일전까지의 이동평균PM_{10}농도	숫자	㎍/㎥
pm10_ma150	si_pm10_ma150	0-150일전까지의 이동평균PM_{10}농도	숫자	㎍/㎥
pm10_ma180	si_pm10_ma180	0-180일전까지의 이동평균PM_{10}농도	숫자	㎍/㎥
pm10_lag1y	si_pm10_lag1y	0-365일전까지의 이동평균PM_{10}농도	숫자	㎍/㎥
pm10_lag2y	si_pm10_lag2y	0-730일전까지의 이동평균PM_{10}농도	숫자	㎍/㎥
pm10_lag3y	si_pm10_lag3y	0-1,095일전까지의 이동평균PM_{10}농도	숫자	㎍/㎥
pm10_lag4y	si_pm10_lag4y	0-1,460일전까지의 이동평균PM_{10}농도	숫자	㎍/㎥
pm10_lag5y	si_pm10_lag5y	0-1,826일전까지의 이동평균PM_{10}농도	숫자	㎍/㎥
PM25	si_PM25	당일의 평균$PM_{2.5}$농도	숫자	㎍/㎥
pm25_lag01	si_pm25_lag01	1일전 평균$PM_{2.5}$농도	숫자	㎍/㎥
pm25_lag02	si_pm25_lag02	2일전 평균$PM_{2.5}$농도	숫자	㎍/㎥
pm25_lag03	si_pm25_lag03	3일전 평균$PM_{2.5}$농도	숫자	㎍/㎥
pm25_lag04	si_pm25_lag04	4일전 평균$PM_{2.5}$농도	숫자	㎍/㎥
pm25_lag05	si_pm25_lag05	5일전 평균$PM_{2.5}$농도	숫자	㎍/㎥
pm25_lag06	si_pm25_lag06	6일전 평균$PM_{2.5}$농도	숫자	㎍/㎥
pm25_lag07	si_pm25_lag07	7일전 평균$PM_{2.5}$농도	숫자	㎍/㎥
pm25_lag08	si_pm25_lag08	8일전 평균$PM_{2.5}$농도	숫자	㎍/㎥

Volume: 43, Article ID: e2021015

https://doi.org/10.4178/epih.e2021015

pm25_lag09	si_pm25_lag09	9일전 평균$PM_{2.5}$농도	숫자	㎍/㎥
pm25_lag10	si_pm25_lag10	10일전 평균$PM_{2.5}$농도	숫자	㎍/㎥
pm25_lag11	si_pm25_lag11	11일전 평균$PM_{2.5}$농도	숫자	㎍/㎥
pm25_lag12	si_pm25_lag12	12일전 평균$PM_{2.5}$농도	숫자	㎍/㎥
pm25_lag13	si_pm25_lag13	13일전 평균$PM_{2.5}$농도	숫자	㎍/㎥
pm25_lag14	si_pm25_lag14	14일전 평균$PM_{2.5}$농도	숫자	㎍/㎥
pm25_ma30	si_pm25_ma30	0-30일전까지의 이동평균$PM_{2.5}$농도	숫자	㎍/㎥
pm25_ma60	si_pm25_ma60	0-60일전까지의 이동평균$PM_{2.5}$농도	숫자	㎍/㎥
pm25_ma90	si_pm25_ma90	0-90일전까지의 이동평균$PM_{2.5}$농도	숫자	㎍/㎥
pm25_ma120	si_pm25_ma120	0-120일전까지의 이동평균$PM_{2.5}$농도	숫자	㎍/㎥
pm25_ma150	si_pm25_ma150	0-150일전까지의 이동평균$PM_{2.5}$농도	숫자	㎍/㎥
pm25_ma180	si_pm25_ma180	0-180일전까지의 이동평균$PM_{2.5}$농도	숫자	㎍/㎥
pm25_lag1y	si_pm25_lag1y	0-365일전까지의 이동평균$PM_{2.5}$농도	숫자	㎍/㎥
pm25_lag2y	si_pm25_lag2y	0-730일전까지의 이동평균$PM_{2.5}$농도	숫자	㎍/㎥
pm25_lag3y	si_pm25_lag3y	0-1,095일전까지의 이동평균$PM_{2.5}$농도	숫자	㎍/㎥
pm25_lag4y	si_pm25_lag4y	0-1,460일전까지의 이동평균$PM_{2.5}$농도	숫자	㎍/㎥

Volume: 43, Article ID: e2021015

https://doi.org/10.4178/epih.e2021015

pm25_lag5y	si_pm25_lag5y	0-1,826일전까지의 이동평균$PM_{2.5}$농도	숫자	㎍/㎥
SO2	si_SO2	당일의 평균SO_2농도	숫자	ppm
so2_lag01	si_so2_lag01	1일전 평균SO_2농도	숫자	ppm
so2_lag02	si_so2_lag02	2일전 평균SO_2농도	숫자	ppm
so2_lag03	si_so2_lag03	3일전 평균SO_2농도	숫자	ppm
so2_lag04	si_so2_lag04	4일전 평균SO_2농도	숫자	ppm
so2_lag05	si_so2_lag05	5일전 평균SO_2농도	숫자	ppm
so2_lag06	si_so2_lag06	6일전 평균SO_2농도	숫자	ppm
so2_lag07	si_so2_lag07	7일전 평균SO_2농도	숫자	ppm
so2_lag08	si_so2_lag08	8일전 평균SO_2농도	숫자	ppm
so2_lag09	si_so2_lag09	9일전 평균SO_2농도	숫자	ppm
so2_lag10	si_so2_lag10	0일전 평균SO_2농도	숫자	ppm
so2_lag11	si_so2_lag11	1일전 평균SO_2농도	숫자	ppm
so2_lag12	si_so2_lag12	2일전 평균SO_2농도	숫자	ppm
so2_lag13	si_so2_lag13	3일전 평균SO_2농도	숫자	ppm
so2_lag14	si_so2_lag14	4일전 평균SO_2농도	숫자	ppm
so2_ma30	si_so2_ma30	0-30일전까지의 이동평균SO_2농도	숫자	ppm
so2_ma60	si_so2_ma60	0-60일전까지의 이동평균SO_2농도	숫자	ppm
so2_ma90	si_so2_ma90	0-90일전까지의 이동평균SO_2농도	숫자	ppm

Volume: 43, Article ID: e2021015

https://doi.org/10.4178/epih.e2021015

so2_ma120	si_so2_ma120	0-120일전까지의 이동평균SO_2농도	숫자	ppm
so2_ma150	si_so2_ma150	0-150일전까지의 이동평균SO_2농도	숫자	ppm
so2_ma180	si_so2_ma180	0-180일전까지의 이동평균SO_2농도	숫자	ppm
so2_lag1y	si_so2_lag1y	0-365일전까지의 이동평균SO_2농도	숫자	ppm
so2_lag2y	si_so2_lag2y	0-730일전까지의 이동평균SO_2농도	숫자	ppm
so2_lag3y	si_so2_lag3y	0-1,095일전까지의 이동평균SO_2농도	숫자	ppm
so2_lag4y	si_so2_lag4y	0-1,460일전까지의 이동평균SO_2농도	숫자	ppm
so2_lag5y	si_so2_lag5y	0-1,826일전까지의 이동평균SO_2농도	숫자	ppm
NO2	si_NO2	당일의 평균NO_2농도	숫자	ppm
no2_lag01	si_no2_lag01	1일전 평균NO_2농도	숫자	ppm
no2_lag02	si_no2_lag02	2일전 평균NO_2농도	숫자	ppm
no2_lag03	si_no2_lag03	3일전 평균NO_2농도	숫자	ppm
no2_lag04	si_no2_lag04	4일전 평균NO_2농도	숫자	ppm
no2_lag05	si_no2_lag05	5일전 평균NO_2농도	숫자	ppm
no2_lag06	si_no2_lag06	6일전 평균NO_2농도	숫자	ppm
no2_lag07	si_no2_lag07	7일전 평균NO_2농도	숫자	ppm
no2_lag08	si_no2_lag08	8일전 평균NO_2농도	숫자	ppm

Volume: 43, Article ID: e2021015

https://doi.org/10.4178/epih.e2021015

no2_lag09	si_no2_lag09	9일전 평균NO_2농도	숫자	ppm
no2_lag10	si_no2_lag10	10일전 평균NO_2농도	숫자	ppm
no2_lag11	si_no2_lag11	11일전 평균NO_2농도	숫자	ppm
no2_lag12	si_no2_lag12	12일전 평균NO_2농도	숫자	ppm
no2_lag13	si_no2_lag13	13일전 평균NO_2농도	숫자	ppm
no2_lag14	si_no2_lag14	14일전 평균NO_2농도	숫자	ppm
no2_ma30	si_no2_ma30	0-30일전까지의 이동평균NO_2농도	숫자	ppm
no2_ma60	si_no2_ma60	0-60일전까지의 이동평균NO_2농도	숫자	ppm
no2_ma90	si_no2_ma90	0-90일전까지의 이동평균NO_2농도	숫자	ppm
no2_ma120	si_no2_ma120	0-120일전까지의 이동평균NO_2농도	숫자	ppm
no2_ma150	si_no2_ma150	0-150일전까지의 이동평균NO_2농도	숫자	ppm
no2_ma180	si_no2_ma180	0-180일전까지의 이동평균NO_2농도	숫자	ppm
no2_lag1y	si_no2_lag1y	0-365일전까지의 이동평균NO_2농도	숫자	ppm
no2_lag2y	si_no2_lag2y	0-730일전까지의 이동평균NO_2농도	숫자	ppm
no2_lag3y	si_no2_lag3y	0-1,095일전까지의 이동평균NO_2농도	숫자	ppm
no2_lag4y	si_no2_lag4y	0-1,460일전까지의 이동평균NO_2농도	숫자	ppm

Volume: 43, Article ID: e2021015

https://doi.org/10.4178/epih.e2021015

no2_lag5y	si_no2_lag5y	0-1,826일전까지의 이동평균NO_2농도	숫자	ppm
CO	si_CO	당일의 평균CO농도	숫자	ppm
co_lag01	si_co_lag01	1일전 평균CO농도	숫자	ppm
co_lag02	si_co_lag02	2일전 평균CO농도	숫자	ppm
co_lag03	si_co_lag03	3일전 평균CO농도	숫자	ppm
co_lag04	si_co_lag04	4일전 평균CO농도	숫자	ppm
co_lag05	si_co_lag05	5일전 평균CO농도	숫자	ppm
co_lag06	si_co_lag06	6일전 평균CO농도	숫자	ppm
co_lag07	si_co_lag07	7일전 평균CO농도	숫자	ppm
co_lag08	si_co_lag08	8일전 평균CO농도	숫자	ppm
co_lag09	si_co_lag09	9일전 평균CO농도	숫자	ppm
co_lag10	si_co_lag10	10일전 평균CO농도	숫자	ppm
co_lag11	si_co_lag11	11일전 평균CO농도	숫자	ppm
co_lag12	si_co_lag12	12일전 평균CO농도	숫자	ppm
co_lag13	si_co_lag13	13일전 평균CO농도	숫자	ppm
co_lag14	si_co_lag14	14일전 평균CO농도	숫자	ppm
co_ma30	si_co_ma30	0-30일전까지의 이동평균CO농도	숫자	ppm
co_ma60	si_co_ma60	0-60일전까지의 이동평균CO농도	숫자	ppm
co_ma90	si_co_ma90	0-90일전까지의 이동평균CO농도	숫자	ppm

Volume: 43, Article ID: e2021015

https://doi.org/10.4178/epih.e2021015

co_ma120	si_co_ma120	0-120일전까지의 이동평균CO농도	숫자	ppm
co_ma150	si_co_ma150	0-150일전까지의 이동평균CO농도	숫자	ppm
co_ma180	si_co_ma180	0-180일전까지의 이동평균CO농도	숫자	ppm
co_lag1y	si_co_lag1y	0-365일전까지의 이동평균CO농도	숫자	ppm
co_lag2y	si_co_lag2y	0-730일전까지의 이동평균CO농도	숫자	ppm
co_lag3y	si_co_lag3y	0-1,095일전까지의 이동평균CO농도	숫자	ppm
co_lag4y	si_co_lag4y	0-1,460일전까지의 이동평균CO농도	숫자	ppm
co_lag5y	si_co_lag5y	0-1,826일전까지의 이동평균CO농도	숫자	ppm
O3	si_O3	당일의 평균O_3농도	숫자	ppm
o3_lag01	si_o3_lag01	1일전 평균O_3농도	숫자	ppm
o3_lag02	si_o3_lag02	2일전 평균O_3농도	숫자	ppm
o3_lag03	si_o3_lag03	3일전 평균O_3농도	숫자	ppm
o3_lag04	si_o3_lag04	4일전 평균O_3농도	숫자	ppm
o3_lag05	si_o3_lag05	5일전 평균O_3농도	숫자	ppm
o3_lag06	si_o3_lag06	6일전 평균O_3농도	숫자	ppm
o3_lag07	si_o3_lag07	7일전 평균O_3농도	숫자	ppm
o3_lag08	si_o3_lag08	8일전 평균O_3농도	숫자	ppm

Volume: 43, Article ID: e2021015

https://doi.org/10.4178/epih.e2021015

o3_lag09	si_o3_lag09	9일전 평균O_3농도	숫자	ppm
o3_lag10	si_o3_lag10	10일전 평균O_3농도	숫자	ppm
o3_lag11	si_o3_lag11	11일전 평균O_3농도	숫자	ppm
o3_lag12	si_o3_lag12	12일전 평균O_3농도	숫자	ppm
o3_lag13	si_o3_lag13	13일전 평균O_3농도	숫자	ppm
o3_lag14	si_o3_lag14	14일전 평균O_3농도	숫자	ppm
o3_ma30	si_o3_ma30	0-30일전까지의 이동평균O_3농도	숫자	ppm
o3_ma60	si_o3_ma60	0-60일전까지의 이동평균O_3농도	숫자	ppm
o3_ma90	si_o3_ma90	0-90일전까지의 이동평균O_3농도	숫자	ppm
o3_ma120	si_o3_ma120	0-120일전까지의 이동평균O_3농도	숫자	ppm
o3_ma150	si_o3_ma150	0-150일전까지의 이동평균O_3농도	숫자	ppm
o3_ma180	si_o3_ma180	0-180일전까지의 이동평균O_3농도	숫자	ppm
o3_lag1y	si_o3_lag1y	0-365일전까지의 이동평균O_3농도	숫자	ppm
o3_lag2y	si_o3_lag2y	0-730일전까지의 이동평균O_3농도	숫자	ppm
o3_lag3y	si_o3_lag3y	0-1,095일전까지의 이동평균O_3농도	숫자	ppm
o3_lag4y	si_o3_lag4y	0-1,460일전까지의 이동평균O_3농도	숫자	ppm

Volume: 43, Article ID: e2021015

https://doi.org/10.4178/epih.e2021015

o3_lag5y	si_o3_lag5y	0-1,826일전까지의<br>이동평균O_3농도	숫자	ppm
