# Supplementary material for: Establishment of the Korea National Health and Nutrition Examination Survey air pollution study dataset for the researchers on the health impact of ambient air pollution
Source: Epidemiol Health. 2021 Feb 8;43:e2021015. doi: 10.4178/epih.e2021015 (PMC8060520; doi:10.4178/epih.e2021015)
Supplement: Supplementary Material 2. — Exposure level of moving average of 0 to 30 days of ambient air pollutants during the study period (2007-2017) [file epih-43-e2021015-suppl2.pdf]

Volume: 43, Article ID: e2021015

https://doi.org/10.4178/epih.e2021015

**Supplementary Material 2.** Exposure level of moving average of 0 to 30 days of ambient air pollutants during the study period (2007-2017)

| | **Mean** | **SD** | **Min** | **Percentile** | | | **Max** | **IQR** |
|---|---|---|---|---|---|---|---|---|
| | | | | **25th** | **50th** | **75th** | | |
| Air pollutants | | | | | | | | |
| Sigungu | | | | | | | | |
| $PM_{10}$ (μg/$m^3$) | 49.2 | 132.9 | 23.1 | 40.2 | 48.8 | 57.1 | 87.6 | 16.9 |
| $PM_{2.5}$ (μg/$m^3$) | 24.9 | 33.5 | 9.7 | 20.6 | 24.5 | 28.9 | 46.2 | 8.3 |
| $NO_2$ (ppb) | 23.4 | 0.1 | 1.8 | 14.7 | 22.5 | 31.8 | 53.3 | 17.1 |
| CO (ppb) | 483.2 | 23.6 | 152.7 | 377.8 | 458.5 | 562.8 | 1546.1 | 185.0 |
| $SO_2$ (ppb) | 4.8 | 0.0 | 0.8 | 3.3 | 4.4 | 5.8 | 18.9 | 2.5 |
| $O_3$ (ppb) | 24.9 | 0.0 | 8.0 | 17.9 | 24.3 | 31.6 | 62.9 | 13.7 |
| Geo-code | | | | | | | | |
| $PM_{10}$ (μg/$m^3$) | 49.3 | 138.6 | 22.2 | 40.1 | 48.7 | 57.3 | 97.7 | 17.2 |
| $PM_{2.5}$ (μg/$m^3$) | 24.9 | 35.5 | 9.9 | 20.6 | 24.6 | 29.1 | 51.4 | 8.5 |
| $NO_2$ (ppb) | 23.4 | 0.1 | 1.4 | 14.9 | 22.3 | 32.0 | 53.5 | 17.1 |
| CO (ppb) | 483.9 | 24.7 | 129.1 | 375.6 | 458.1 | 563.3 | 1607.2 | 187.7 |
| $SO_2$ (ppb) | 4.8 | 0.0 | 0.6 | 3.3 | 4.4 | 5.9 | 28.9 | 2.6 |
| $O_3$ (ppb) | 24.9 | 0.0 | 8.1 | 17.7 | 24.2 | 31.6 | 64.1 | 13.9 |

SD, standard deviation; IQR, interquartile range.
