# Supplementary material for: Establishment of the Korea National Health and Nutrition Examination Survey air pollution study dataset for the researchers on the health impact of ambient air pollution
Source: Epidemiol Health. 2021 Feb 8;43:e2021015. doi: 10.4178/epih.e2021015 (PMC8060520; doi:10.4178/epih.e2021015)
Supplement: Supplementary Material 3. — Exposure level of moving average of 0 to 90 days of ambient air pollutants during the study period (2007-2017) [file epih-43-e2021015-suppl3.pdf]

Volume: 43, Article ID: e2021015

https://doi.org/10.4178/epih.e2021015

Supplementary Material 3. Exposure level of moving average of 0 to 90 days of ambient air pollutants during the study period (2007-2017)

	Mean	SD	Min	Percentile			Max	IQR
				25th	50th	75th		
Air pollutants								
Sigungu								
PM_{10} (μg/m^3)	49.2	96.5	23.8	41.4	48.6	56.1	83.1	14.7
$PM_{2.5}$ (μg/m^3)	24.9	23.6	10.1	21.3	24.7	28.0	41.4	6.7
NO_2 (ppb)	23.3	0.1	1.7	14.8	22.3	31.8	47.1	17.0
CO (ppb)	481.8	19.5	185.8	386.5	48534.0	561.2	1472.3	174.7
SO_2 (ppb)	4.8	0.0	0.9	3.5	4.4	5.9	15.8	2.4
O_3 (ppb)	25.1	0.0	9.6	18.7	24.6	31.2	49.8	12.5
Geo-code								
PM_{10} (μg/m^3)	49.3	100.7	22.0	41.5	48.5	56.2	86.8	14.7
$PM_{2.5}$ (μg/m^3)	24.9	25.4	9.2	21.4	24.7	28.1	43.3	6.7
NO_2 (ppb)	23.4	0.1	0.2	15.1	22.6	31.6	47.8	16.5
CO (ppb)	482.5	20.4	167.3	384.7	458.8	563.7	1518.8	179.0
SO_2 (ppb)	4.8	0.0	0.8	3.5	4.4	5.9	27.7	2.4
O_3 (ppb)	25.1	0.1	9.6	18.6	24.7	31.3	49.5	12.7

SD, standard deviation; IQR, interquartile range.
