# Supplementary material for: Establishment of the Korea National Health and Nutrition Examination Survey air pollution study dataset for the researchers on the health impact of ambient air pollution
Source: Epidemiol Health. 2021 Feb 8;43:e2021015. doi: 10.4178/epih.e2021015 (PMC8060520; doi:10.4178/epih.e2021015)
Supplement: Supplementary Material 4. — Exposure level of moving average of 0 to 180 days of ambient air pollutants during the study period (2007-2017) [file epih-43-e2021015-suppl4.pdf]

Volume: 43, Article ID: e2021015

https://doi.org/10.4178/epih.e2021015

Supplementary Material 4. Exposure level of moving average of 0 to 180 days of ambient air pollutants during the study period (2007-2017)

	Mean	SD	Min	Percentile			Max	IQR
				25th	50th	75th		
Air pollutants								
Sigungu								
PM_{10} (μg/m^3)	49.5	65.5	26.6	43.5	49.4	54.7	80.3	11.2
$PM_{2.5}$ (μg/m^3)	25.0	16.3	12.5	22.3	24.9	27.3	39.6	5.0
NO_2 (ppb)	23.3	0.1	1.8	15.6	22.3	31.7	45.7	16.1
CO (ppb)	483.2	13.4	184.1	401.7	473.4	563.3	1022.9	161.6
SO_2 (ppb)	4.8	0.0	1.2	3.7	4.6	5.7	15.8	2.0
O_3 (ppb)	25.2	0.0	11.5	20.2	25.0	30.0	44.9	9.8
Geo-code								
PM_{10} (μg/m^3)	49.6	69.7	26.6	43.4	49.3	54.9	83.5	11.5
$PM_{2.5}$ (μg/m^3)	25.0	18.0	10.4	22.2	24.9	27.5	41.2	5.3
NO_2 (ppb)	23.5	0.1	2.2	15.7	22.6	31.8	45.8	16.1
CO (ppb)	483.6	14.0	173.0	399.0	474.1	562.6	1191.5	163.6
SO_2 (ppb)	4.9	0.0	1.0	3.7	4.6	5.7	24.5	2.0
O_3 (ppb)	25.2	0.0	11.5	20.1	24.9	29.7	46.4	9.6

SD, standard deviation; IQR, interquartile range.
