# Supplementary material for: Establishment of the Korea National Health and Nutrition Examination Survey air pollution study dataset for the researchers on the health impact of ambient air pollution
Source: Epidemiol Health. 2021 Feb 8;43:e2021015. doi: 10.4178/epih.e2021015 (PMC8060520; doi:10.4178/epih.e2021015)
Supplement: Supplementary Material 5. — Exposure level of moving average of 0 to 365 days of ambient air pollutants during the study period (2007-2017) [file epih-43-e2021015-suppl5.pdf]

Volume: 43, Article ID: e2021015

https://doi.org/10.4178/epih.e2021015

Supplementary Material 5. Exposure level of moving average of 0 to 365 days of ambient air pollutants during the study period (2007-2017)

	Mean	SD	Min	Percentile			Max	IQR
				25th	50th	75th		
Air pollutants								
Sigungu								
PM_{10} (μg/m^3)	49.9	39.3	29.8	46.3	49.2	54.2	69.4	7.9
$PM_{2.5}$ (μg/m^3)	25.2	11.1	13.5	23.1	25.0	27.1	36.2	4.0
NO_2 (ppb)	23.6	0.1	2.6	16.7	21.7	32.7	41.1	16.0
CO (ppb)	490.4	8.7	202.8	419.2	506.5	560.0	805.6	140.8
SO_2 (ppb)	4.9	0.0	1.3	4.0	4.7	5.5	14.3	1.5
O_3 (ppb)	24.8	0.0	17.1	22.2	24.6	27.1	41.0	4.9
Geo-code								
PM_{10} (μg/m^3)	50.1	43.8	26.5	46.2	49.3	54.5	71.9	8.3
$PM_{2.5}$ (μg/m^3)	25.2	12.9	11.2	22.9	25.1	27.3	37.0	4.4
NO_2 (ppb)	23.7	0.1	2.1	16.8	22.3	32.4	41.6	15.6
CO (ppb)	490.7	9.1	162.5	420.5	505.5	558.4	831.9	137.9
SO_2 (ppb)	4.9	0.0	1.1	4.0	4.8	5.5	23.6	1.5
O_3 (ppb)	24.7	0.0	17.1	22.2	24.5	27.0	44.5	4.8

SD, standard deviation; IQR, interquartile range.
