# Supplementary material for: Establishment of the Korea National Health and Nutrition Examination Survey air pollution study dataset for the researchers on the health impact of ambient air pollution
Source: Epidemiol Health. 2021 Feb 8;43:e2021015. doi: 10.4178/epih.e2021015 (PMC8060520; doi:10.4178/epih.e2021015)
Supplement: Supplementary Material 6. — Exposure level of moving average of 0 to 1,095 days of ambient air pollutants during the study period (2007-2017) [file epih-43-e2021015-suppl6.pdf]

Volume: 43, Article ID: e2021015

https://doi.org/10.4178/epih.e2021015

Supplementary Material 6. Exposure level of moving average of 0 to 1,095 days of ambient air pollutants during the study period (2007-2017)

	Mean	SD	Min	Percentile			Max	IQR
				25th	50th	75th		
Air pollutants								
Sigungu								
PM_{10} (μg/m^3)	50.9	38.9	31.0	47.0	50.2	55.1	71.1	8.1
$PM_{2.5}$ (μg/m^3)	25.4	9.7	14.3	23.8	25.1	27.0	37.1	3.2
NO_2 (ppb)	23.8	0.1	3.2	16.9	21.9	32.9	40.4	16.0
CO (ppb)	499.2	8.7	241.1	424.2	521.0	568.9	772.6	144.7
SO_2 (ppb)	5.0	0.0	1.6	4.2	4.8	5.6	13.9	1.4
O_3 (ppb)	24.3	0.0	16.6	21.7	24.2	26.5	37.0	4.8
Geo-code								
PM_{10} (μg/m^3)	51.0	43.0	28.2	47.1	50.3	55.5	73.4	8.4
$PM_{2.5}$ (μg/m^3)	25.5	11.2	12.6	23.7	25.2	27.2	38.4	3.5
NO_2 (ppb)	23.9	0.1	2.5	17.3	22.3	32.8	40.5	15.5
CO (ppb)	499.7	9.1	200.0	428.0	520.2	570.0	844.8	142.0
SO_2 (ppb)	5.1	0.0	1.2	4.2	4.8	5.6	24.5	1.4
O_3 (ppb)	24.3	0.0	16.5	21.7	24.2	26.4	40.0	4.7

SD, standard deviation; IQR, interquartile range.
