# Supplementary material for: Establishment of the Korea National Health and Nutrition Examination Survey air pollution study dataset for the researchers on the health impact of ambient air pollution
Source: Epidemiol Health. 2021 Feb 8;43:e2021015. doi: 10.4178/epih.e2021015 (PMC8060520; doi:10.4178/epih.e2021015)
Supplement: Supplementary file 7 [file epih-43-e2021015-suppl7.docx]

**Data profile**

**Title:** An establishment of the Korea National Health and Nutrition Examination Survey air pollution study dataset for the researchers on the health impact of ambient air pollution

**국문제목:** 대기오염 건강영향 연구를 위한 국민건강영양조사 대기질 통합 데이터베이스 구축

|  |
| --- |
| Myung-Jae Hwang^1^, Jisun Sung^1^, Miryoung Yoon^1^, Jong-Hun Kim^1^, Hui-Young Yun^2^, Dae-Ryun Choi^2^, Youn-Seo Koo^2^, Kyungwon Oh^3^, Sungha Yun^3^, Hae-Kwan Cheong^1^*  ^1^Department of Social and Preventive Medicine, Sungkyunkwan University School of Medicine, Suwon, Gyeonggi-do, Republic of Korea  ^2^Department of Environmental and Energy Engineering, Anyang University, Anyang, Gyeonggi-do, Republic of Korea  ^3^Division of Health and Nutrition Survey and Analysis, Bureau of Chronic Disease Prevention and Control, Korea Disease Control and Prevention Agency, Cheongju, Chungcheongbuk-do, Korea |
|  |

Address for correspondence: Hae-Kwan Cheong, M.D., Dr.P.H.

Department of Social and Preventive Medicine, Sungkyunkwan University School of Medicine

2066 Seobu-ro, Jangan-gu, Suwon, Gyeonggi-do 16419, Republic of Korea

E-mail: hkcheong@skku.edu

**Abstract**

To provide a nationwide representative dataset for the study on health impact of air pollution, we combined the data from the Korea National Health and Nutrition Examination Survey with the daily air quality and weather data by matching the date of examination and the residential address of the participants. The database of meteorological factors and air quality as sources of exposure data were estimated using the Community Multiscale Air Quality model. The linkage dataset was merged by three ways; administrative district, *si-gun-gu* (city, county, and district), and geocode (in latitude and longitude coordinate units) based on the participants’ residential address, respectively. During the study period, the exposure dataset of 85,018 individuals (38,306 men and 46,712 women) whose examination dates were recorded were obtained. According to the definition of exposure period, the dataset was combined with the data on short-term, mid-term, and long-term exposure to air pollutants and the meteorological indices. Calculation of the daily merged dataset’s average air pollution linked by *si-gun-gu* and geocode units showed similar results. This study generated a daily average of meteorological indices and air pollution exposure dataset for all regions including rural and remote areas in Korea for 11 years. It is expected to provide a platform for the researchers studying the health impact of air pollution and climate change on the representative population and area, which may facilitate the establishment of local health care plans by understanding the residents’ health status at the local as well as national level.

**Keywords:** Korea National Health and Nutrition Examination Survey (KNHANES), air pollution, particulate matter, health impact, data profile

**Introduction**

세계적으로 연간 420만 명의 조기사망을 초래하는 대기오염은 공중보건의 주요 위험요인 중 하나로 부각되고 있다 [1]. 우리나라는 1960년대 이후 급격한 도시화와 산업화를 거치면서 도시의 대기오염이 악화된 바 있다. 대기오염으로 인한 건강 영향에 관한 연구는 국내에서 1990년대 후반부터 보고되어 왔으나 대부분의 연구가 서울특별시 혹은 대도시를 중심으로 수행되었으며 이로 인하여 농촌 지역을 포함하여 전국을 대상으로 한 대기오염으로 인한 건강영향 관련 연구는 부족한 실정이다. 미국이나 중국 등의 국가에 비해 국토가 좁고 인구밀도가 높으며 대기오염의 영향이 전국적으로 미치는 우리나라의 현실을 감안할 때, 대기오염 건강영향 연구는 전국을 단위로 지역 간 인구학적, 사회경제적 차이를 고려하여 연구할 수 있어야 한다.

최근 국내 전국 지역 수준의 대기 오염 측정망이 빠른 속도로 늘어나고 있지만, 아직 대도시나 수도권을 제외한 지역의 측정망은 부족하다 [2-4]. 특히 최근 문제가 되고 있는 초미세먼지(PM_2.5_)는 수도권 지역을 중심으로 측정되어 왔기 때문에 전국을 대상으로건강 영향 연구를 하는 데에 큰 제한점으로 작용하고 있다.

질병관리청에서는 국민건강영양조사, 한국인유전체역학조사 사업 코호트 자료 등 체계적인 조사를 전국적 단위로 건강 데이터베이스(database, DB)를 주기적으로 생산하고 있다 [6]. 이 DB들은 전국적인 대표성을 갖추고 있을 뿐 아니라 지역 간 격차를 볼 수 있도록 다양한 정보를 갖추고 있을 뿐 아니라 엄격한 질관리시스템을 적용하고 있어 세계적 기준으로도 매우 우수한 건강연구를 위한 자료원을 제공하고 있다. 이러한 DB는 대기오염 자료와 결합할 경우 대기오염 건강영향 연구에 즉시 활용할 수 있는 장점이 있다 [5]. 이를 위해서는 실질적으로 대기오염 노출자료 연계를 통해 연구가 수행되어야 한다. 국가 수준에서 기상 및 대기 DB와 건강 DB를 결합한 연구 수행을 위해서는 두 종류의 자료 간의 시공간적 결합이 가능하도록 호환성이 확보되어야 하고, 시공간 해상도와 자료의 범위가 일치해야 한다.

그러나 실제에 있어서는 연구자들이 대기오염 노출에 따른 건강영향을 평가하기 위해서는 정확한 노출 일자(조사 일자, 검진 일자 등), 당일의 대기 농도 자료를 필요로 하고, 조사 대상자의 시·군·구 이상 단위의 거주지 주소 정보가 필요하지만 이에 대한 자료원을 확보하지 못하여 연구를 수행하는 데 어려움이 있다. 개인정보 보호의 측면에서 이들 건강DB들은 저해상도 자료만 제공되기 때문에 개별 연구자가 각 대상자의 시간-공간정보를 이용하여 대기오염자료나 기상자료와 결합하는 것은 매우 어렵다. 이러한 한계점을 극복하고 효율적인 데이터 활용을 위해서는 기존 건강DB와 환경DB를 기관이 직접 연계하여 통합 DB를 생성하고 결합된 자료를 비실명화하고 시간공간자료를 저해상도로 변환한 후 연구자에게 분양하는 방법을 적용할 경우 개별 연구자들이 직접 개인정보나 시간-공간정보에 접근하지 않고도 자료를 다양한 역학연구에 활용할 수 있다.

본 사업에서는 체계적인 방법으로 대기오염 DB 등 환경 위해 요인 DB를 생산하여 국민건강영향조사와 연계한 DB를 생성하였고, 연구자들의 연구를 위한 DB 플랫폼을 제공하여 선진적인 연구 활성화와 지역 수준의 건강정책 수립의 과학적 근거를 생성하는데 활용되게 하고자 한다.

**Data resource**

국민건강영양조사는 1995년 제정된 국민건강증진법 제16조에 근거하여 시행하는 전국 규모의 조사이다. 1998년부터 2005년까지 3년 주기로 시행하였으며, 2007년부터는 매년 시행하고 있다. 본 조사는 국민의 건강 수준, 건강 관련 의식 및 행태, 식품 및 영양 섭취 실태에 대한 국가 및 시도 단위의 대표성과 신뢰성을 갖춘 통계를 산출하고 있다(Table 1) [6,7].

**Table 1.** Survey components of the Korea National Health and Nutrition Examination Survey from 2007 to 2017

| **Survey** | **Components** |
| --- | --- |
| Health interview | Household characteristics, socioeconomic status (e.g., education, household income, and occupational status), economic status, medical use, vaccination, medical conditions, activity limitation, quality of life, injury, smoking, alcohol use, physical activity, mental health, oral health, safety, weight control, and reproductive health for women |
| Health examination | Body measurement, blood pressure and pulse rate, blood test, urine test, muscle strength test, lung function, dental examination, otolaryngeal and ophthalmologic examination |
| Nutrition survey | Dietary behavior, dietary supplement use, households with food security, food frequency, and food intake |

건강설문조사와 검진 조사는 이동 검진센터에서 실시하며 영양조사는 대상 가구를 직접 방문하여 시행한다. 건강 설문조사의 교육 및 경제활동, 이환, 의료이용 항목, 영양조사의 전체 항목은 면접 방법으로 조사하였으며, 건강 설문조사 항목 중 흡연, 음주 등 건강 행태 영역을 자기 기입식으로 조사하였다. 검진 조사는 직접 계측, 관찰, 검체 분석 등의 방법으로 수행하였다.

국민건강영양조사의 표본 추출 틀은 표본 설계 시점에서 가용한 가장 최근 시점의 인구주택 총 조사 자료를 사용하고, 현재 모집단 특성을 반영할 수 있는 최신 정보 사용을 위해 공동주택공시가격 자료를 추가하여 기본 추출 틀을 보완하고 모집단 포함율을 향상시켰다. 이를 통해 목표 모집단인 대한민국에 거주하는 만 1세 이상 국민에 대하여 대표성 있는 표본을 추출할 수 있도록 한다. 국민건강영양조사의 기본 추출틀은 인구주택 총조사 자료이지만, 제5기(2010-2012)는 설계시점에서 인구주택총조사 자료가 노후화되어 주민등록 인구 및 아파트단지시세조사 자료를 추출틀로 사용하였다[7].

**Population coverage**

본 사업에서는 2007년부터 2017년까지 조사에 참여한 대상자들 중 검진 조사 일자가 기입된 대상자 총 85,018명을 대상으로 노출자료를 연계하였다. 연도별 조사 대상자 수의 분포를 관찰한 결과, 국민건강영양조사 제4기에 해당하는 2007년에는 7~12월 조사를 실시하여 4,246명으로 가장 적었고, 2009년 10,078명으로 가장 많았다. 연구기간동안 17개 시도별 분포는 경기도가 16,181명으로 가장 많았고, 2016년 새로 추가된 행정구역인 세종특별자치시가 330명으로 가장 적었다 (Table 2).

**Table 2.** Distribution of survey participants of the 2007-2017 Korea National Health and Nutrition Examination Survey (KNHAES) by 17 regions^1^

| **Year** | **2007** | **2008** | | **2009** | **2010** | **2011** | | **2012** | **2013** | **2014** | | **2015** | **2016** | **2017** | | **Total** |  |  |  |  |
| --- | --- | --- | --- | --- | --- | --- | --- | --- | --- | --- | --- | --- | --- | --- | --- | --- | --- | --- | --- | --- |
| **Regions** |  | | | | | | | | | | | | | | | |  |  |  |  |
| Metropolitan city |  |  | |  |  |  | |  |  |  | |  |  |  | |  |  |  |  |  |
| Seoul | 708 | 1,543 | | 1,680 | 1,705 | 1,647 | | 1,618 | 1,455 | 1,335 | | 1,313 | 1,526 | 1,484 | | 16,014 |  |  |  |  |
| Busan | 241 | 596 | | 633 | 482 | 497 | | 425 | 388 | 423 | | 348 | 500 | 479 | | 5,012 |  |  |  |  |
| Daegu | 245 | 427 | | 467 | 408 | 384 | | 384 | 440 | 406 | | 405 | 370 | 368 | | 4,304 |  |  |  |  |
| Incheon | 195 | 564 | | 648 | 483 | 488 | | 442 | 452 | 410 | | 451 | 419 | 477 | | 5,029 |  |  |  |  |
| Gwangju | 160 | 395 | | 348 | 234 | 258 | | 237 | 229 | 218 | | 235 | 244 | 253 | | 2,811 |  |  |  |  |
| Daejeon | 142 | 301 | | 354 | 277 | 223 | | 249 | 270 | 299 | | 252 | 265 | 255 | | 2,887 |  |  |  |  |
| Ulsan | 88 | 249 | | 254 | 198 | 201 | | 196 | 164 | 145 | | 136 | 144 | 172 | | 1,947 |  |  |  |  |
| Sejong^2^ | NA | NA | | NA | NA | NA | | NA | NA | NA | | NA | 164 | 166 | | 330 |  |  |  |  |
| Province |  | |  | |  | |  | |  | |  | |  | |  | |  |  |  |  |
| Gyeonggi-do | 854 | 1,930 | | 2,104 | 2,003 | 1,845 | | 1,713 | 1,906 | 1,781 | | 1,660 | 201 | 184 | | 16,181 |  |  |  |  |
| Gangwon-do | 148 | 290 | | 341 | 257 | 221 | | 221 | 249 | 251 | | 290 | 1,830 | 1,804 | | 5,902 |  |  |  |  |
| Chungcheongbuk-do | 122 | 333 | | 348 | 236 | 272 | | 194 | 214 | 231 | | 198 | 220 | 252 | | 2,620 |  |  |  |  |
| Chungcheongnam-do | 226 | 419 | | 514 | 368 | 325 | | 310 | 316 | 282 | | 316 | 259 | 207 | | 3,542 |  |  |  |  |
| Jeollabuk-do | 135 | 406 | | 351 | 328 | 316 | | 309 | 245 | 222 | | 257 | 315 | 239 | | 3,123 |  |  |  |  |
| Jeollanam-do | 220 | 481 | | 472 | 321 | 313 | | 304 | 206 | 187 | | 220 | 252 | 294 | | 3,270 |  |  |  |  |
| Gyeongsangbuk-do | 342 | 601 | | 672 | 518 | 429 | | 459 | 438 | 393 | | 377 | 263 | 253 | | 4,745 |  |  |  |  |
| Gyeongsangnam-do | 308 | 549 | | 624 | 475 | 456 | | 425 | 421 | 427 | | 374 | 387 | 386 | | 4,832 |  |  |  |  |
| Jeju-do | 112 | 223 | | 268 | 180 | 172 | | 154 | 178 | 157 | | 145 | 444 | 436 | | 2,469 |  |  |  |  |
| **Total** | 4,246 | 9,307 | | 10,078 | 8,473 | 8,047 | | 7,640 | 7,571 | 7,167 | | 6,977 | 7,803 | 7,709 | | 85,018 |  |  |  |  |

NA, not applicable.

^1^Evaluation of the socio-demographic characteristics showed that the women participants outnumbered the men participants by 9.8% point (women: n=46,712, 54.9% and men: n=38,306, 45.1%). The group aged 50-60 years had the highest number of participants (n=12,107), followed by those aged 40-50 years and those aged 30-40 years. Over 1,151 participants did not respond to the question related to income level. Meanwhile, 23,981 participants responded to the question related to the income level: “above 2 million Korean won and below 3 million Korean won.” A total of 3,495 participants did not respond to the question related to education level, while 32,138 responded that they only finished “elementary school.”

^2^Survey was included in the survey in 2016.

조사에 참여한 대상자들의 인구사회학적 특성을 관찰한 결과(각 항목의 결측치 포함) 남성 38,306명(45.1%), 여성 46,712명(54.9%)으로 여성의 참여자 비율이 9.8% 더 높았다. 연령별 대상자는 50세 이상 60세 미만의 인구가 12,107명으로 가장 많았고, 다음으로 40세 이상 50세 미만, 30세 이상 40세 미만 순이었다. 자기기입식으로 응답한 소득 수준에 관한 문항의 무응답자 수는 총 1,151명이었다. 대상자들의 소득 수준은 ‘200만원 초과 300만원 이하’에 응답한 대상자의 수가 23,981명으로 가장 많았다. 교육 수준에 관한 문항의 무응답자 수는 총 3,495명이었고, 응답한 대상자 중 ‘초등학교 졸업’에 해당하는 대상자가 32,138명으로 가장 많았다 (Table 3).

**Table 3.** Demographic characteristics of participants from 2007 to 2017 Korea National Health and Nutrition Examination Survey (KNHANES)

| **Characteristics** | **Men** | | **Women** | | **Total** |
| --- | --- | --- | --- | --- | --- |
| Total | 38,306 (45.1) | | 46,712 (54.9) | | 85,018 |
| Age (yr) |  |  |  |  |  |
| 1-9 | 5,340 (6.3) | | 4,920 (5.8) | | 10,260 |
| 10-19 | 5,286 (6.2) | | 4,797 (5.6) | | 10,083 |
| 20-29 | 3,106 (3.7) | | 4,095 (4.8) | | 7,201 |
| 30-39 | 4,891 (5.8) | | 6,764 (8.0) | | 11,655 |
| 40-49 | 5,285 (6.2) | | 6,811 (8.0) | | 12,096 |
| 50-59 | 5,152 (6.1) | | 6,955 (8.2) | | 12,107 |
| 60-69 | 4,866 (5.6) | | 6,097 (7.2) | | 10,963 |
| ≥70 | 4,380 (5.1) | | 6,273 (7.4) | | 10,653 |
| Income level (unit: 10 thousand won) |  |  |  |  |  |
| ≤100 | 6,036 (7.0) | | 8,909 (10.6) | | 14,945 |
| 100-200 | 9,668 (11.4) | | 11,833 (13.9) | | 21,501 |
| 200-300 | 11,126 (13.1) | | 12,855 (15.1) | | 23,981 |
| >300 | 10,993 (12.9) | | 12,447 (14.6) | | 23,440 |
| Missing | 483 (0.6) | | 668 (0.8) | | 1,151 |
| Education level |  |  |  |  |  |
| Elementary school | 13,279 (15.6) | | 18,859 (22.2) | | 32,138 |
| Middle school | 4,436 (5.1) | | 4,922 (5.8) | | 9,358 |
| High school | 9,654 (11.4) | | 11,442 (13.5) | | 21,096 |
| Over university | 9,241 (10.9) | | 9,690 (11.4) | | 18,931 |
| Missing | 1,696 (2.0) | | 1,799 (2.1) | | 3,495 |

Values are presented as number (%) or number.

**Measurements**

**Exposure data**

본 사업에서 기상 및 대기 DB는 Community Multiscale Air Quality (CMAQ)을 이용한 화학수송모델링을 통해 생성하였다. 시간 단위와 격자 단위에 따라 바람장, 온·습도장을 비롯하여 3차원으로 생성된 기상자료는 배출량 모델링과 화학수송 모델링의 입력자료로 이용되고, 배출량 모델링은 기상자료와 각 배출원의 특성을 고려하여 대기질 모델링에 적용할 수 있는 화학종·시간·공간별 배출량을 생성한다.

화학수송모델은 기상자료의 기상인자와 배출량 모델에서 산정된 화학종·시간·공간별 배출량을 이용하여 가스와 에어로졸 화학반응 및 이류확산 방정식을 수치적으로 해석한 후 시·공간적으로 오염 물질 농도를 산정하게 된다. 생성된 기상모델은 CMAQ을 통해 생성된 재분석 기상자료를 입력으로 하여 Weather Research Forecast version 3.6.1을 사용하였고, 배출량 생성 모델은 Sparse Matrix Operator Kernel Emissions version 2.7을 사용하였다 [8].

산정된 기상 자료는 평균 기온, 평균 습도, 평균 강수량, 평균 풍향, 평균 일사량, 평균 지표압력으로 3 km 단위로 생산되었다. 대기질 자료는 미세먼지(PM_10_), 초미세먼지(PM_2.5_)는 1 km^2^ (1 km x 1 km) 단위로, 가스상물질인 이산화질소 (NO_2_), 일산화탄소 (CO), 이산화황 (SO_2_), 오존(O_3_)은 9 km^2^ (3 km x 3 km) 단위로 산출되었다. 생성된 자료는 기본적으로 위·경도 좌표(geocode)로 생성되고, 격자 단위의 자료를 시·군·구 지역 경계에 따라 가중합으로 재산정하여 지역 단위의 노출 자료가 산정되었다.

본 자료에서는 인공위성관측 Aerosol Optical Depth (AOD) 자료와 다중회귀분석 (MLR, Multiple Linear Regression) 방법을 활용하여 PM_10_, PM_2.5_, O_3_의 재분석 결과를 보완하고 검증하였다. 특히 2013년 이전에는 PM_2.5_의 측정 자료가 부족하기 때문에 화학수송모델링 수행결과를 근거로 재분석 자료를 산출하여 보완하였다. 본 사업에서 재분석한 모델링 자료와 실제 국내 관측지점에서의 측정치와의 상관성과 타당도 검증을 수행하였다. 그 결과, PM_10_과 PM_2.5_의 R^2^는 각각 0.81, 0.64였다 [8].

**Definition of exposure period**

일반적으로 대기오염으로 인한 질환 및 사망의 발생 및 악화에 있어서 연관성은 시계열 연구 (time-series study)를 통해 영향력을 확인할 수 있다 [9,10]. 시계열 자료를 통해 대기오염의 노출에 따른 건강 영향을 평가할 때, 노출 기간에 따라 크게 단기(short-term), 중기(mid-term), 장기(long-term)의 영향을 관찰할 수 있다. 이러한 건강 영향은 노출 기간에 따라 달라지는데, 단기노출로 인해 체내로 유입된 대기 중 유해물질은 체내 대사 메커니즘에 급성 영향을 미치고, 지속적인 노출(장기노출)은 질환의 발생 및 이환에 영향을 미쳐 사망까지 이르게 한다.

따라서 본 사업에서는 국민건강영양조사에서 제공하고 있는 다양한 건강 자료원의 활용을 위해 일별로 생산된 대기오염 DB는 노출 기간에 따라 산정하여 연계하였다. 현재 대기오염의 노출 기간 정의에 따른 명확한 기준은 없으나, 현재까지 국내외에서 보고되고 있는 환경유해인자 노출로 인한 건강 영향 연구들에 기반하여 정의하였다(Figure 1).

1. **Definition of short-term exposure**

여러 역학 연구에서는 단기간 높은 농도의 대기오염 물질 노출로 인한 건강영향을 평가하고 있다. 기존의 연구들은 건강 자료원의 제한으로 인해 대기오염으로 인한 단기 효과를 평가하였다면, 최근에는 다양한 자료원의 접근이 용이해짐에 따라 대기오염의 장기노출로 인한 건강 영향에 관한 연구들이 활발하게 보고되고 있다[11,12,13].

본 사업에서는 노출자료와 건강조사 자료가 동시에 가용한 2007년부터 2017년까지 국민건강영양조사에 참여한 조사 대상자들의 검진일자를 이용하여 대기오염의 단기노출 자료를 연계하였다. 대기오염의 지연효과(lag effect)를 고려하여 조사 당일, 1일전(lag 01), 2일전(lag 02), … , 14일 전(lag14)까지의 일별 평균 기상 및 대기 자료를 연계하였다.

1. **Definition of mid-term exposure**

일반적으로 대기오염의 노출 효과는 단기노출과 장기노출로 구분되지만, 최근 검진 조사의 혈액 내 다양한 생체지표의 활용으로 인해 대기오염의 중기노출로 인한 건강 영향 연구도 보고되고 있다. 예를 들어, 혈액 내 정상적인 적혈구의 수명은 120일 정도이고, 당화혈색소는 60-90일 정도임을 고려하면, 대기오염의 노출로 인한 혈액 내 생체 지표의 변화를 관찰하기 위해 중기노출을 적용하는 것이 적절하다[14].

본 자료에서는 중기노출에 대한 자료는 지연효과를 고려하여 기상 및 대기 자료에 대해조사 당일로부터 30일 전까지, 60일 전까지, 90일 전까지, 120일 전까지, 150일 전까지, 180일 전까지의 일별 이동평균(moving average)를 적용하여 산출하였다.

1. **Definition of long-term exposure**

일반적으로 대기오염의 장기노출은 연(year) 단위 노출 농도로 산정한다. 이는 대기오염의 지속적인 노출로 인한 건강영향을 평가하기 위해 장기간 추적 관찰하는 코호트연구 (cohort study) 뿐만 아니라 국민건강영양조사, 지역사회건강조사와 같이 다년간 시행된 단면조사(cross-sectional study)에서도 활용할 수 있다[2,15].

본 자료에서는 조사 대상자들의 검진 일자를 이용하여 지연효과를 고려하여 기상 및 대기 자료에 대해 조사 당일로부터 365일 전까지(1년), 730일 전까지(2년), 1,095일 전까지(3년), 1,460일 전까지(4년), 1,826일 전까지(5년)의 일별 이동평균(moving average)를 적용하여 산출하였다. 5년 전까지의 이동평균 산출 자료는 노출기간 중 1년이 366일을 감안하여 산정하였다.

**Merging method**

본 사업에서 국민건강영양조사와 기상 및 대기 DB의 연계는 조사 대상자의 검진일자 기준으로 결합하였다. 연계 자료는 조사 대상자의 거주지 주소에 기반하여 시·군·구 단위로 결합된 자료와 위·경도 좌표(geocode)를 기준으로 결합된 자료로 구분된다(Figure 2) (Supplementary Material 1). 이 두가지 노출 해상도에 따른 변수를 검진일자 변수와 연결하여 임의의 linkage key를 생성하여 연계하였다.

Table 4는 2007년부터 2017년까지 산출된 기상 및 대기 DB를 이용하여 국민건강영양조사 대상자들의 거주지 주소를 기반으로 결합한 일평균 노출 자료의 분포이다. 시·군·구 단위로 결합된 자료와 위경도 좌표로 결합된 자료 모두 대기오염 물질의 평균 노출은 비슷한 수준이었다. 그러나 몇몇 대기오염 물질에 대한 일평균 최대 노출 수준은 위·경도 좌표 단위로 결합된 자료에서 높았다. 중기 및 장기 노출 수준에 대한 결과는 Supplement Materials 2-6에 제시하였다.

**Table 4.** Daily exposure level of ambient air pollutants during the study period (2007-2017)

|  | **Mean** | **SD** | **Min** | **Percentile** | | | **Max** | **IQR** |
| --- | --- | --- | --- | --- | --- | --- | --- | --- |
|  |  |  |  | **25th** | **50th** | **75th** |  |  |
| Air pollutants |  |  |  |  |  |  |  |  |
| *Si-gun-gu* |  |  |  |  |  |  |  |  |
| PM_10_ (μg/m^3^) | 49.6 | 21.6 | 10.9 | 34.6 | 45.9 | 59.8 | 260.8 | 25.2 |
| PM_2.5_ (μg/m^3^) | 25.2 | 11.9 | 2.2 | 16.6 | 23.2 | 31.4 | 95.3 | 14.8 |
| NO_2_ (ppb) | 24.4 | 13.8 | 0.3 | 13.6 | 21.8 | 32.6 | 98.3 | 19.0 |
| CO (ppb) | 489.4 | 219.1 | 90.8 | 341.6 | 439.7 | 571.8 | 1841.4 | 230.2 |
| SO_2_ (ppb) | 4.9 | 2.7 | 0.3 | 3.1 | 4.3 | 6.1 | 30.1 | 2.9 |
| O_3_ (ppb) | 24.7 | 10.7 | 0.8 | 16.5 | 23.6 | 32.3 | 72.7 | 15.8 |
| Geocode |  |  |  |  |  |  |  |  |
| PM_10_ (μg/m^3^) | 49.8 | 21.9 | 10.4 | 34.4 | 46.0 | 60.0 | 259.5 | 25.6 |
| PM_2.5_ (μg/m^3^) | 25.3 | 12.1 | 0.9 | 16.6 | 23.1 | 31.5 | 90.7 | 14.9 |
| NO_2_ (ppb) | 24.5 | 14.0 | 0.3 | 13.7 | 21.8 | 32.8 | 101.9 | 19.1 |
| CO (ppb) | 490.8 | 223.4 | 88.4 | 342.6 | 442.2 | 575.4 | 1808.8 | 232.8 |
| SO_2_ (ppb) | 5.0 | 3.0 | 0.1 | 3.1 | 4.3 | 6.1 | 44.5 | 2.9 |
| O_3_ (ppb) | 24.7 | 10.8 | 0.8 | 16.4 | 23.7 | 32.5 | 76.2 | 16.1 |

SD, standard deviation; Min, minimum; Max, maximum; IQR, interquartile range; PM_10_, particulate matter; PM_2.5_, ultrafine particulate matter; NO_2_, nitrogen dioxide; CO, carbon monoxide; SO_2_, sulfur dioxide; O_3_, ozone; ppb, parts per billion.

**Strengths and weaknesses**

본 사업은 대기오염으로 인한 건강영향 연구를 위해 국내 건강 자료의 활용을 증진하고자 국민건강영양조사와 기상 및 대기 DB를 연계하였다. 기존의 연구들은 국내에서 생산된 건강자료를 이용하여 대기오염 노출과의 연구 분석을 시행할 시, 측정점 자료가 존재하지 않은 지역에 대한 제한점으로 인해 주로 8개 대도시 지역 위주로 연구 결과가 산출되었다. 이러한 한계를 극복하기 위해 본 사업에서는 국내 전 지역에 대한 기상 및 대기오염 노출자료를 생산하고, 도시뿐만 아니라 농촌 지역에 거주하는 인구를 대상으로 국민건강영양조사와 같이 국내 건강자료원을 연계하여 분석을 가능하게 함으로써 전 국민의 대표성을 갖는 연구 결과가 도출될 수 있는 기반을 마련하였다. 또한 국민건강영양조사는 연중 시행하는 조사이므로 계절적인 편중이 없는 장기간에 걸친 자료를 구축할 수 있었다. 이에 따라 시간적, 공간적 대표성이 확보되었다. 특히, 개인의 수준에서 건강행태학적 요인(흡연력, 음주력, 신체활동 등)에 대한 정보를 제공하고 있어 대기오염 노출로 인한 질환 발생에 기여하는 요인들을 고려하여 건강 위험을 평가할 수 있다. 이러한 분석을 가능하게 하여 각 지자체에서는 거주민의 건강 영향에 관한 관심이 크게 증하게 되고, 이러한 관심은 각 지자체의 지역 보건의료계획에도 반영되는 계기가 될 것으로 예상된다. 그러나 본 사업에서 산출한 기상 및 대기질 DB는 모델링 자료로써 실제 관측치와의 차이에 대한 제한점은 여전히 남아 있고, 비관측 지점에 대한 불확실성을 완전히 극복하는 것을 어려울 것으로 판단된다.

국민건강영양조사는 매년 설문 조사를 통해 보건의료와 관련된 다양한 지표들을 제공하고 있고, 정밀한 검진 조사를 통해 국가 수준의 건강 지표를 생산하고 있다. 이러한 국내의 대표적인 조사 자료와 대기오염 노출 자료를 연계하여 역학연구자들의 효율적인 정보 활용을 위해 data profile을 작성하였다. 이 profile을 통해 보다 다양한 분야에서 자료가 활용된다면, 이는 우리나라 지역의 인구 및 사회경제학적 특성을 고려한 대기오염의 건강영향 연구를 통해 지자체 수준에서 위해성 기반 대기오염 관련 정책을 기획하고 수행할 수 있는 이론적 근거 자료로 활용될 수 있을 것으로 기대된다.

**Data accessibility**

2007년부터 2017년까지 조사된 국민건강영양조사와 연계된 기상 및 대기오염 DB는 연계자료 제공 절차에 따라 제공받을 수 있다 (http://knhanes.cdc.go.kr). 연계자료는 연구자의 연구계획서를 심의한 후 학술연구자료처리실 등을 통해 제공된다.

**Acknowledgement**

This work was supported by the Research Program funded by the Korea Centers for Disease Control and Prevention (2019-ER7106-00).

**Conflict of interest**

The authors have no conflicts of interest to declare for this study.

**Figure 1.** Definition of exposure period in this study.

**Figure 2.** Merging the method for air pollution database and Korea National Health and Nutrition Examination Survey.

**Reference**

1. World Health Organization. Ambient air pollution: A global assessment of exposure and burden of disease. 2016.
2. Kim H, Byun G, Choi Y, Kim S, Kim SY, Lee JT. Effects of long-term exposure to air pollution on all-cause mortality and cause-specific mortality in seven major cities of South Korea: Korean national health and nutritional examination surveys with mortality follow-up. Environ Res 2021;192:110290.
3. Han MH, Yi HJ, Kim YS, Kim YS. Effect of seasonal and monthly variation in weather and air pollution factors on stroke incidence in Seoul, Korea. Stroke 2015;46:927-935.
4. Son JY, Lee JT, Kim H, Yi O, Bell ML. Susceptibility to air pollution effects on mortality in Seoul, Korea: a case-crossover analysis of individual-level effect modifiers. J Expo Sci Environ Epidemiol 2012;22:227-234.
5. Dabass A, Talbott EO, Venkat A, Rager J, Marsh GM, et al. Association of exposure to particulate matter (PM2.5) air pollution and biomarkers of cardiovascular disease risk in adult NHANES participants (2001–2008). Int J Hyg Environ Health 2016;219:301-310.
6. http://knhanes.cdc.go.kr.
7. Kweon S, Kim Y, Jang MJ, Kim Y, Kim K, Choi S, et al. Data resource profile: the Korea national health and nutrition examination survey (KNHANES). Int J Epidemiol 2014;43:69-77.
8. Koo YS, Choi DR, Yun HY, Yoon GW, Lee JB. A development of PM concentration reanalysis method using CMAQ with surface data assimilation and MAIAC AOD in Korea. Journal of Korean Society for Atmospheric Environment. 2020;36:558-573.
9. Roberts S. Interactions between particulate air pollution and temperature in air pollution mortality time series studies. Environ Res 2004;96:328-337.
10. Zhang C, Ding R, Xiao C, Xu Y, Cheng H, Zhu F, et al. Association between air pollution and cardiovascular mortality in Hefei, China: a time-series analysis. Environ Pollut 2017;229:790-797.
11. Lee JT, Kim H, Hong YC, Kwon HJ, Schwartz J, Christiani DC. Air pollution and daily mortality in seven major cities of Korea, 1991–1997. Environ Res 2000;84:247-254.
12. Park AK, Hong YC, Kim H. Effect of changes in season and temperature on mortality associated with air pollution in Seoul, Korea. J Epidemiol Community Health 2011;65:368-375.
13. Kim OJ, Kim SY, Kim H. Association between long-term exposure to particulate matter air pollution and mortality in a South Korean National Cohort: comparison across different exposure assessment approaches. Int J Environ Res Public Health 2017;14(10):1103.
14. Hwang MJ, Kim JH, Koo YS, Yun HY, Cheong HK. Impacts of ambient air pollution on glucose metabolism in Korean adults: a Korea National Health and Nutrition Examination Survey study. Environ Health 2020;19:1-11.
15. Shin J, Choi J, Kim KJ. Association between long-term exposure of ambient air pollutants and cardiometabolic diseases: a 2012 Korean community health survey. Nutr Met Cardiovasc Dis 2019;29:144-151.
